# NLRP3 inflammasome in acute kidney injury: molecular mechanisms, post-translational regulation, and immunotherapeutic potential

**DOI:** 10.3389/fimmu.2026.1924802

**Published:** 2026-08-18

**Authors:** Mingru Ma, Manling Zhang, Hongfei Sun, Xinran Xie, Xudong Guo, Hongbin Li, Yong Jin

**Affiliations:** 1 Inner Mongolia Key Laboratory for Pathogenesis and Diagnosis of Rheumatic and Autoimmune Diseases, The Affiliated Hospital of Inner Mongolia Medical University, Hohhot, China; 2 The State Key Laboratory of Reproductive Regulation and Breeding of Grassland Livestock, College of Life Science, Inner Mongolia University, Hohhot, China; 3 Department of Rheumatology and Immunology, The Affiliated Hospital of Inner Mongolia Medical University, Hohhot, China; 4 School of Basic Medicine Sciences, Inner Mongolia Medical University, Hohhot, China

**Keywords:** acute kidney injury, molecular mechanisms, NLRP3 inflammasome, post-translational modifications, targeted therapy

## Abstract

Acute kidney injury (AKI) is a critical clinical syndrome characterized by a rapid decline in renal function. Dysregulated inflammatory responses play a crucial role in its pathogenesis, contributing to high patient mortality and a tendency to progress to chronic kidney disease (CKD). As a crucial sensor of the innate immune system, the NLRP3 inflammasome participates in the occurrence and development of AKI and its transition to CKD by activating caspase-1, promoting the maturation of IL-1β/IL-18, and inducing pyroptosis. This review elucidates the molecular composition of the NLRP3 inflammasome, its canonical and non-canonical activation pathways, and, for the first time, integrates the critical roles of post-translational modifications—including ubiquitination, phosphorylation, acetylation, SUMOylation, and palmitoylation—in regulating its activation, as well as their specific activation mechanisms across distinct subtypes of AKI. The specific mechanisms and pathological significance of the NLRP3 inflammasome in ischemia-reperfusion, sepsis-associated, and contrast-induced AKI were analyzed. Furthermore, we summarize current interventional strategies targeting the NLRP3 inflammasome and its upstream/downstream molecules, including direct inhibitors, mitophagy inducers, K^+^ channel modulators, caspase-1 inhibitors, and indirect inhibitor-based agents. Moreover, we assess their protective effects in AKI animal models and the bottlenecks in clinical translation. Finally, future research directions are proposed, such as cell type-specific functional analysis, dynamic regulation of post-translational modifications, and combined targeted therapy for the long-term transition of AKI to CKD, aiming to provide a theoretical basis and novel ideas for the precise therapy of AKI.

## 1 Introduction

Acute kidney injury (AKI) is a clinical syndrome characterized by a rapid loss of renal function caused by various etiologies. Essentially, it involves a sudden decline in excretory function and significantly increases the risk of patients progressing to chronic kidney disease (CKD) and end-stage renal disease (ESRD) (1). AKI is often accompanied by a multitude of severe metabolic disorders, including elevated blood urea nitrogen levels, electrolyte abnormalities, metabolic acidosis, and fluid imbalance. Clinical studies indicated that the primary causes of hospital-acquired AKI encompass ischemic injury, drug toxicity, and sepsis (2). In recent years, the incidence of AKI has been continuously rising. Globally, approximately 13.3 million people suffer from AKI annually, with about 1.7 million dying from AKI and its associated complications. Therefore, actively exploring targeted therapeutic strategies for AKI and delaying its progression to CKD has emerged as a crucial topic in the field of nephrology (3, 4).

Inflammation and immune dysregulation play a central role in the complex pathogenesis of AKI. The Nod-like receptor family pyrin domain containing 3 (NLRP3) inflammasome is one of the most extensively studied multi-protein complexes. Its typical components include the NLRP3 protein, the apoptosis-associated speck-like protein containing a caspase recruitment domain (ASC), and the precursor of cysteine-aspartic protease 1 (pro-caspase-1) (5). The activation of the NLRP3 inflammasome typically requires two sequential steps: priming and activation. During the priming phase, inflammatory stimuli such as pathogen-associated molecular patterns (PAMPs) or damage-associated molecular patterns (DAMPs) activate the nuclear factor-κB (NF-κB) signaling pathway via toll-like receptors (TLRs), thereby upregulating the transcription and expression of NLRP3, ASC, pro-caspase-1, pro- IL-1β, and pro-IL-18 (6). In the activation phase, various stimuli (e.g., intracellular potassium efflux, lysosomal disruption, or mitochondrial dysfunction) induce the assembly of the NLRP3 inflammasome, subsequently recruiting ASC and pro-caspase-1 to form a stable complex (7). Activated caspase-1 cleaves pro- IL-1β and pro-IL-18 into their mature forms and promotes their secretion, and cleaves gasdermin D (GSDMD), thereby inducing pyroptosis—a highly pro-inflammatory mode of cell death. Furthermore, recent studies have revealed that the NLRP3 inflammasome can also be activated through non-canonical pathways and alternative activation pathways, further enriching its regulatory network.

Substantial evidence indicates that the NLRP3 inflammasome plays a critical role in the initiation and progression of AKI. In the pathological state of AKI, the NLRP3 inflammasome can be activated not only in classical immune cells (e.g., macrophages) (8), but also in renal parenchymal cells, particularly non-immune renal tubular epithelial cells and mesangial cells, following stimulation by ischemia, toxins, or sepsis (9). This aberrant activation triggers the release of pro-inflammatory cytokines (e.g, IL-1β and IL-18), exacerbates the local inflammatory response in the kidney, promotes renal tubular injury, pyroptosis, and interstitial fibrosis, thereby facilitating the transition from AKI to CKD (10).

In summary, as a key molecular hub connecting innate immunity and kidney injury, the NLRP3 inflammasome exhibits a significant pathogenic role in various types of AKI. Based on the existing research evidence, this review systematically delineated the composition and activation mechanisms of the NLRP3 inflammasome, subsequently analyzed its specific roles in AKI induced by different etiologies, and then explore the intrinsic relationship between the NLRP3 inflammasome and AKI. Finally, we evaluated the potential strategies and prospects of targeting the NLRP3 inflammasome in the treatment of AKI. The unique contribution of this review lies in that it for the first time integrates and analyzes the association between post-translational modification regulatory networks, and the specific activation mechanisms of NLRP3 across different AKI subtypes, aiming to provide a novel theoretical basis and intervention concepts for the clinical treatment of AKI.

## 2 Overview of the NLRP3 inflammasome

The NLRP3 inflammasome is a crucial component of the innate immune system. It has the ability to detect danger signals both inside and outside the cell and assemble into high-molecular-weight protein complex, thereby mediating inflammatory responses and cell death. The following sections will elaborate on its components, their respective functions, and primary biological roles.

### 2.1 Components of the NLRP3 inflammasome and their respective roles

**Figure 1:**
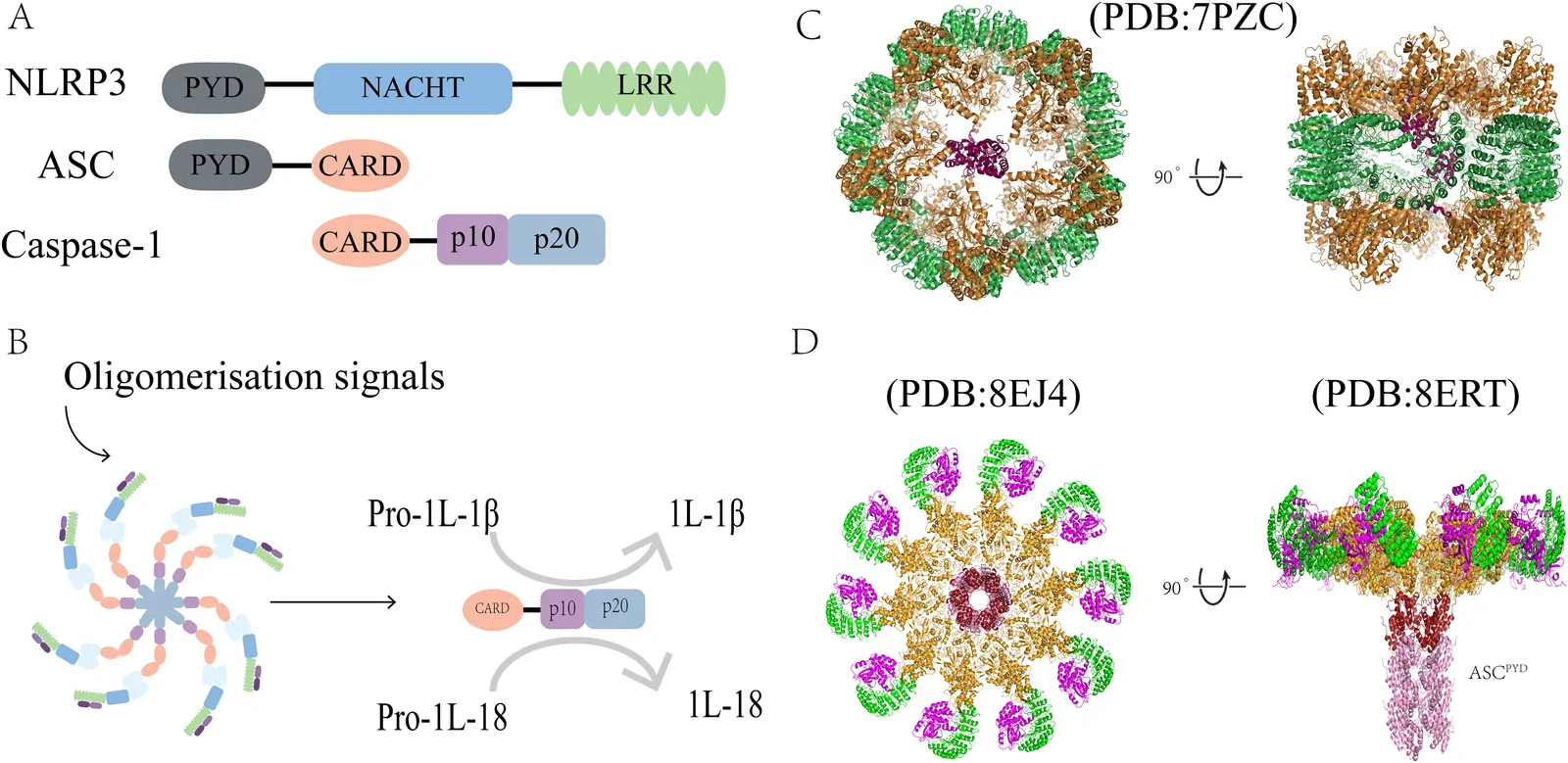
Structure of the NLRP3 inflammasome. **(A)** Domain composition of the NLRP3 inflammasome complex. An illustration of the sensor-adaptor-effector mechanism of NLRP3, which depicts the interaction of NLRP3 with ASC through the PYD domain, and the interaction of ASC with caspase-1 through the CARD domain. **(B)** The activation of the oligomeric NLRP3 inflammasome facilitates the activation of caspase-1 and the maturation of IL-1β and IL-18. **(C)** The closed and inactive cage-like structure of NLRP3 (PDB: 7PZC), presented with a top view (left) and a side view (right). **(D)** Schematic representation of the active NLRP3 inflammasome disc-like structure (including ASCPYD; PDB: 8EJ4, PDB: 8ERT), shown with a top view (left) and a side view (right).

The assembly of the NLRP3 inflammasome is an ordered signaling cascade. Its core components can be classified into three categories: sensor, adaptor, and effector, corresponding to the NLRP3 protein, apoptosis-associated speck-like protein containing a CARD (ASC), and the cysteine-aspartic protease 1 (caspase-1) (11) (**Figure 1A**).

The sensor NLRP3 is a tripartite intracellular pattern recognition receptor composed of three functional domains: the C-terminal leucine-rich repeat (LRR), the central nucleotide-binding and oligomerization domain with adenosine triphosphate (ATP) catalytic function (NACHT), and an N-terminal pyrin domain (PYD) (12). In the resting state, the NACHT domain of NLRP3 binds to adenosine diphosphate (ADP), and the LRR domain folds back onto the NACHT domain, inhibiting NLRP3 self-oligomerization, thereby maintaining its inactive conformation. The specific NLRP3 inhibitor MCC950 (also known as CP-456,773; CRID3) can bind non-covalently between the NACHT domain and the LRR domain, significantly stabilizing the autoinhibitory conformation (13). The results of cryo-electron microscopy show that NLRP3 bound to MCC950 exists in the form of a decamer, which was assembled into a cage-like structure by five homodimers (**Figure 1C**). Within this structure, the NACHT domains are located at the apical axis, and the sides are connected by five pairs of LRR domains, while the PYD domains are wrapped within the cage-like structure and cannot bind to ASC. This spatial conformation forms a protective mechanism to prevent the spontaneous activation of NLRP3 (14). Upon receiving activating signals such as PAMPs or DAMPs, NLRP3 undergoes conformational changes, relieving the autoinhibition effect of the LRR domain. Subsequently, the NACHT domain binds and hydrolyzes ATP, and with the assistance of proteins such as NIMA-related kinase 7 (NEK7), promotes NLRP3 self-oligomerization into trimers, exposing the N-terminal PYD domain to recruit the adaptor protein ASC (11). Human NEK7 is a member of the serine/threonine kinase family, which not only plays a crucial role in mitosis but also is an essential regulator for NLRP3 activation (15). The cryo-electron microscopic structure of the NLRP3-NEK7 complex reveals that the binding of NEK7 facilitates the depolymerization of the NLRP3 cage structure and its assembly into an activated disc-like structure (**Figure 1D**). The C- terminal lobe of NEK7 is encircled by the LRR domain of NLRP3. The first half of the C - terminal lobe of NEK7 interacts with the LRR domain, and the second half interacts with the NACHT domain. Meanwhile, ASC forms a PYD - PYD filamentous structure with NLRP3 to further stabilize the assembly of NLRP3 (16).

The adaptor ASC is a two-domain adaptor protein, which contains the N-terminal PYD domain and the C-terminal caspase activation and recruitment domain (CARD) (17). The PYD domain is responsible for homotypic interactions with the PYD domain of NLRP3, whereas the CARD domain is responsible for recruiting the effector pro-caspase-1. When NLRP3 is stimulated to oligomerize, multiple ASC molecules are recruited to the NLRP3 complex via homologous PYD-PYD interactions, and further undergo prion-like aggregation takes place to form long-chain ASC filaments. Multiple ASC filaments can aggregate into a single macromolecular focus, referred to as ASC speck, which function as a signal amplification platform, to significantly enhance the recruitment efficiency of downstream pro-caspase-1 by exposing their C-terminal CARD domains (18).

Caspase-1 is the first identified cysteine-aspartic protease and belongs to the inflammatory caspase family. Its inactive precursor (pro-caspase-1) is composed of an N-terminal CARD domain, a large catalytic subunit in the middle (p20), and a small catalytic subunit at the C-terminal (p10). At the ASC speck, pro-caspase-1 is recruited via CARD-CARD interactions and undergoes dimerization and proximity-induced auto-proteolysis, being cleaved into p20 and p10 subunits, which then assemble into an enzymatically active heterodimer (**Figure 1B**). Activated caspase-1 has dual pro-inflammatory functions: firstly, it can cleave inactive pro- IL-1β and pro-IL-18 into mature forms and promotes their secretion; secondly, it can cleave the pyroptosis-executing protein GSDMD, releasing its N-terminal fragment to perforate the cell membrane, thereby inducing pyroptosis. Through these pathways, caspase-1 serves as a key effector molecule linking inflammatory signals and cell death (19).

### 2.2 Function of the NLRP3 inflammasome

As the core sensor of the innate immune system, the NLRP3 inflammasome performs multiple functions in host immune defense, inflammation regulation, and tissue damage repair through the recognizing of PAMPs and DAMPs. Nevertheless, under pathological conditions, particularly in AKI, its excessive or persistent activation can trigger inflammatory cascades, promotes pyroptosis, and accelerates the process of renal fibrosis. The primary functions are delineated as follows.

#### 2.2.1 Immune defense and inflammatory amplification

In the physiological immune responses, the NLRP3 inflammasome can be activated by PAMPs such as bacterial lipopolysaccharide (LPS) and viral RNA. Subsequently, it releases IL-1β and IL-18, recruits and activates immune cells such as neutrophils and macrophages to clear pathogens. However, in the pathological state of AKI, this protective mechanism is often excessively amplified. For instance, the abnormal activation of NLRP3 in renal interstitial macrophages can lead to the release of the chemokine CCL2, which recruits more monocytes to infiltrate the renal tissue, forms a positive feedback inflammatory loop that exacerbates tubulointerstitial injury (20). Therefore, moderate regulation of NLRP3 activity is crucial for maintaining immune homeostasis and preventing excessive tissue damage.

#### 2.2.2 Mediating pyroptosis and tissue injury

Following the activation of NLRP3 inflammasome, the activated caspase-1 specifically cleaves the GSDMD. Subsequently, its N-terminal fragment forms pores on the cell membrane, leading to cell swelling and rupture, while simultaneously releasing mature IL-1β/IL-18. Interstitial fluid permeates into the cell through these pores causes swelling, disrupting cell membrane integrity and releasing cellular contents such as K^+^, Ca²^+^, and ATP, thereby inducing pyroptosis, a highly pro-inflammatory mode of cell death (21). Studies have shown (22) that in the drug-induced liver injury (DILI) model, pyroptosis and apoptosis driven by the NLRP3-caspase-1-GSDMD pathway interacts and collaborate. This interaction can recruit neutrophils to form an “ inflammatory storm” and significantly exacerbate tissue damage. Similar mechanisms have been observed in ischemic or sepsis-associated AKI, suggesting that targeting this pathway may alleviate renal parenchymal cell loss in AKI (23).

NLRP3 inflammasome-mediated pyroptosis is not solely regulated by the GSDMD protein. Recent studies have revealed a more complex regulatory network: when the canonical pathway is blocked or when specific cell types are stimulated, NLRP3 can shift to Gasdermin E (GSDME)-mediated pyroptosis, which plays a critical role in the pathological progression of various forms of AKI (24). Unlike GSDMD, which is primarily activated by caspase-1 cleavage, GSDME can be cleaved by activated caspase-3, generating an N-terminal active fragment (GSDME-N) that leads to lytic cell death. Shen et al. found in chemotherapy-induced kidney injury models (e.g., cisplatin and doxorubicin) that oxidative stress activates the ERK/JNK pathway, subsequently initiating the caspase-3/GSDME pyroptotic signaling cascade; inhibition of this pathway significantly alleviated renal tubular injury and improved renal function (25). Furthermore, ischemia-reperfusion AKI models have also confirmed that GSDME knockout can inhibit renal tubular pyroptosis and reduce inflammatory infiltration in kidney tissues (26).

#### 2.2.3 Involvement in the AKI-to-CKD transition

The continuous or repeated activation of the NLRP3 inflammasome serves not only as the key driving force for the progression of AKI, but also as one of the core mechanism that promote renal interstitial fibrosis and accelerate the AKI-to-CKD transition. Firstly, IL-1β released upon NLRP3 activation can induce epithelial-mesenchymal transition (EMT) of renal tubular epithelial cellsand increase the deposition of extracellular matrix components (e.g., collagen). Secondly, the NLRP3 signaling pathway can regulate the proliferation and activation of fibroblast, directly exacerbating renal interstitial fibrosis. A recent study further demonstrated that protein lactylation of citrate synthase (CS) can diminish CS activity and mitochondrial function, thereby activating the NLRP3 inflammasome and promoting the AKI-to-CKD transition (27). This finding provides a novel perspective for comprehending the interaction between metabolic remodeling and inflammation and offers a potential intervention target for retarding the progression of AKI.

## 3 Activation and regulatory mechanisms of the NLRP3 inflammasome

The activation of the NLRP3 inflammasome is regulated by a sophisticated and intricate signaling network. Depending on the activation mode, it can be classified into the canonical, non-canonical, and alternative activation pathways. Furthermore, multiple post-translational modifications, including ubiquitination and phosphorylation, also play critical roles in regulating its activity.

### 3.1 Canonical activation pathway of NLRP3

**Figure 2:**
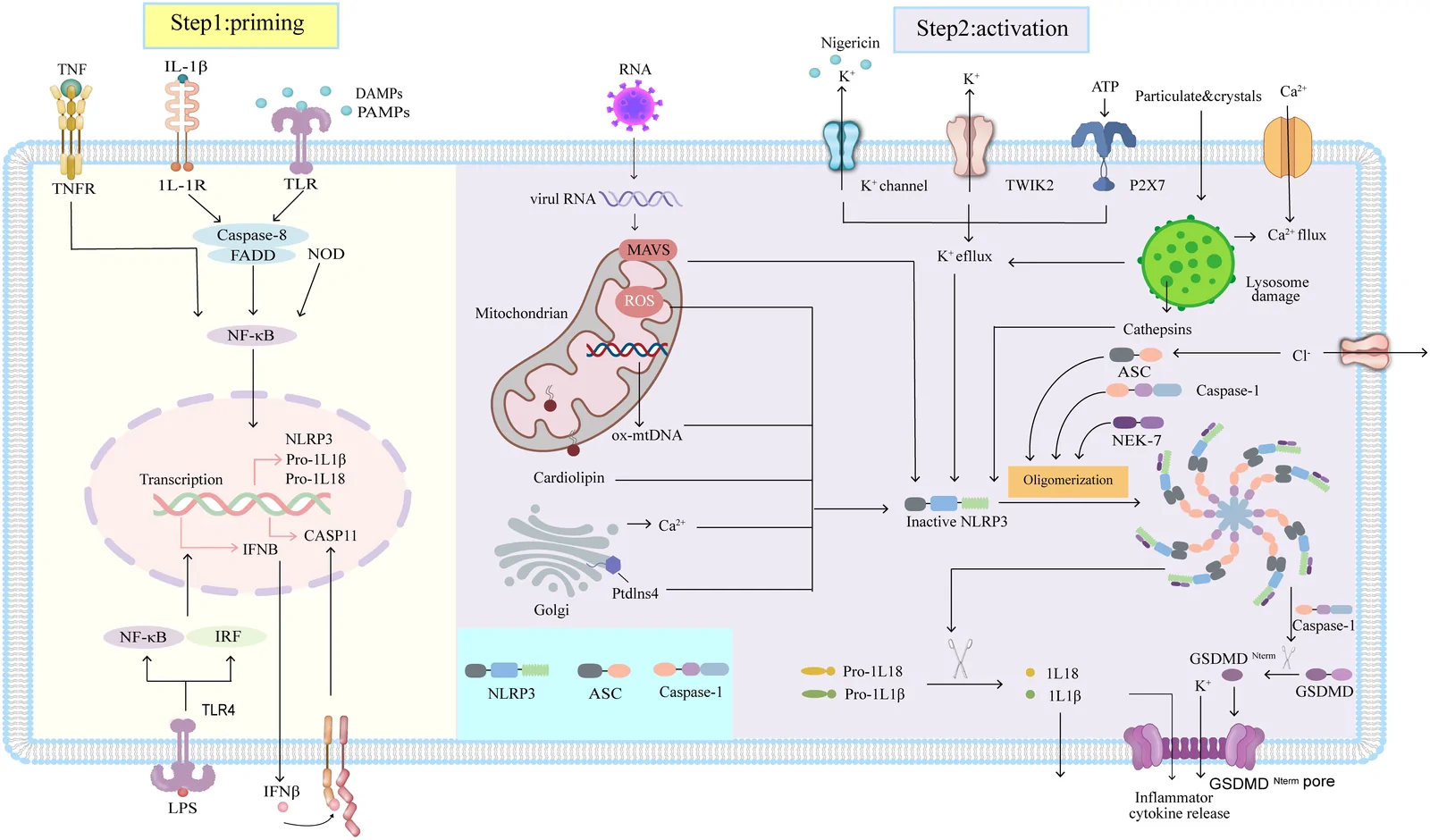
Canonical activation pathway of the NLRP3 inflammasome. The canonical activation pathway of the NLRP3 inflammasome is composed of two stages. During the priming stage (left), Toll-like receptors (TLRs) identify DAMPs or PAMPs and facilitate the transcriptional upregulation of NLRP3 inflammasome components through the NF-κB pathway. In the activation stage (right), activation signals such as viral RNA, fungal hyphae, extracellular ATP, and hyaluronan initiate diverse upstream signaling events, including ion fluxes (K^+^ efflux, Ca²^+^ efflux, and Na^+^ efflux), lysosomal rupture, and mitochondrial dysfunction (mtROS) production, release of Ox-mtDNA. The formation of the NLRP3 inflammasome activates caspase-1, which cleaves pro-IL-1β and pro-IL-18, and gasdermin D (GSDMD), releasing its N-terminal domain that translocates to the cell membrane to form pores, ultimately resulting in pyroptosis.

The canonical activation pathway of the NLRP3 inflammasome comprises two steps (**Figure 2**): priming and activation. During the priming stage, TLRs typically recognize DAMPs or PAMPs. The activation of the downstream pro-inflammatory NF-κB pathway leads to the up-regulation of the transcription and expression of NLRP3, ASC, pro-caspase-1, pro-IL-1β, and pro-IL-18, thereby preparing for the assembly and activation of the inflammasome (28). During this process, the apoptosis-related signaling molecules caspase-8 and Fas-associated death domain protein (FADD) are also participated in the regulation of the upstream signals of the NLRP3 inflammasome. The regulatory functions of caspase-8 and FADD are independent of the classical apoptosis pathway and can also participate in both the transcriptional initiation and post-translational activation of the NLRP3 inflammasome pathway (29). Mechanistically, caspase-8 directly interacts with the inhibitor of κB kinase (IKK) complex via its death effector domain (DED). The IKK complex is a core component for NF-κB signaling pathway activation and can significantly promote the nuclear translocation of NF-κB transcription factors and the transcription of target genes. It also provides necessary signal support for the subsequent activation of NLRP3 inflammasome (30). In general, the priming signal exerts its effects on NLRP3 inflammasome activation mainly reflected in two core aspects: Firstly, through NF-κB pathway-dependent regulation, the expression levels of the NLRP3 receptor protein, pro-caspase-1, and pro- IL-1β are significantly upregulated. Secondly, it is involved in post-translational modifications (PTMs) of the NLRP3 inflammasome, such as ubiquitination, SUMOylation, and phosphorylation (31, 32).

Based on the priming signal, a diverse range of structurally unrelated activation signals (e.g., viral RNA, fungal hyphae, extracellular ATP, hyaluronic acid, reactive oxygen species (ROS), ion fluxes alterations (K^+^ efflux, Ca²^+^ efflux, and Na^+^ efflux), lysosomal rupture, and mitochondrial dysfunction) can initiate NLRP3 oligomerization (33). The activated NLRP3 binds to the C-terminus of the NEK7 protein via its LRR domain, thereby forming the NLRP3-NEK7 complex. Subsequently, NLRP3 recruits ASC through PYD-PYD interactions, which in turn recruits pro-caspase-1 through CARD-CARD interactions, leading to its autocatalytic cleavage and formation of enzymatically active caspase-1. Eventually, the NLRP3-ASC-caspase-1 complex is assembled. Activated caspase-1 can hydrolyze pro- IL-1β and pro-IL-18 to generate the mature pro-inflammatory cytokines IL-1β and IL-18 (34). Several major activation signals and their molecular mechanisms will be emphasized below (**Figure 2**).

#### 3.1.1 Role of K^+^ efflux in NLRP3 inflammasome activation

Early studies indicated that the K^+^ ionophore nigericin induces maturation and secretion of IL-1β in LPS-stimulated mouse macrophages (35). Subsequent studies confirmed that low potassium solution promotes the activation of NLRP3 inflammasome, whereas high potassium solution inhibits its activation by obstructing potassium efflux (36). Various NLRP3 agonists (e.g., nigericin, ATP, granule molecules, and crystals substances) can induce intracellular K^+^ efflux, thereby activating the NLRP3 inflammasome. Among these, K^+^ efflux mediated by the P2X7 receptor channel represents a significant activation pathway (37). When stimulated by ATP, ATP binds to the P2X7 receptor to generate a DAMP signal, and cooperates with the two-pore domain potassium channel TWIK2 (38). The opening of the channel results in transmembrane K^+^ efflux, ultimately activating the NLRP3 inflammasome. Furthermore, K^+^ efflux-mediated NLRP3 inflammasome activation also involves the kinase NEK7, which binds to NLRP3 in the cytoplasm to form a molecular complex, and its catalytic domain interacts with the NLRP3 protein, regulating NLRP3 oligomerization and activation (39). Recent study further revealed that K^+^ efflux is a crucial upstream signal for NLRP3 phase dissociation and activation. By reducing the intracellular K^+^ concentration, regulating ZDHHC7-mediated palmitoylation, and IDR-driven multivalent interactions, NLRP3 liquid-liquid phase separation (LLPS) is triggered to promote ASC recruitment and inflammasome assembly. NLRP3 is activated by caspase-1, followed by the release of IL-1β/IL-18. This study demonstrated that NLRP3 can be activated through ZDHHC7-dependent and K^+^ efflux-dependent activation, ZDHHC7-dependent but K^+^ efflux-independent activation, and ZDHHC7-independent activation (40).

K^+^ efflux serves as a hallmark ionic signaling event that initiates NLRP3 inflammasome assembly and activation, and this pathway has been implicated in the pathogenesis of AKI. Following ischemic insults such as renal ischemia-reperfusion injury, damaged tubular epithelial cells release ATP, which activates membrane-bound P2X7 receptors and sustains K^+^ efflux. Genetic deletion of P2X7R or pharmacological blockade of the receptor abrogates K^+^ efflux, markedly suppresses NLRP3 inflammasome activation, and mitigates ischemia-reperfusion-induced renal tissue injury (41). In cisplatin-induced nephrotoxic AKI models, tubular cytotoxic stimuli promote ATP release and P2X7R channel activation, driving K^+^ efflux that subsequently triggers NLRP3 inflammasome activation, thereby exacerbating intrarenal inflammation and tubular cell death. Notably, NLRP3 deficiency has been shown to ameliorate cisplatin-induced tubular epithelial cell degeneration, detachment, and necrosis, while P2X7R knockout mice exhibit significantly attenuated renal fibrosis (42). Collectively, these findings establish that ATP-P2X7-mediated K^+^ efflux, culminating in NLRP3 inflammasome activation, constitutes a convergent and critical pathogenic axis shared by both ischemic and drug-induced AKI.

#### 3.1.2 Role of lysosomal rupture in NLRP3 inflammasome activation

Lysosomes are organelles containing a variety of hydrolytic enzymes, which are accountable for decomposing biological macromolecules (proteins, nucleic acids, polysaccharides, etc.). Upon stimulation by various damage molecules such as ATP, monosodium urate crystals, silica particles, or bacterial components, the integrity of the lysosomal membrane is disrupted, releasing different cathepsins that participate in NLRP3 inflammasome activation (43). Gaidt et al. found that activation of the cGAS-STING axis in human monocytes causes mitochondrial DNA (mtDNA) escape, leading to lysosomal disruption, K^+^ efflux, and NLRP3 activation. A recent study showed that the ferritin heavy chain (FTH) promotes the activation of NLRP3 inflammasome via ICAM-1 on the surface of hepatic stellate cells (HSCs) through clathrin-mediated endocytosis and lysosomal membrane permeabilization (LMP) (44). In this pathway, the permeability of the lysosomal membrane serves as the core link connecting the FTH-ICAM-1 signaling and NLRP3 activation, suggesting that liver fibrosis can exacerbate chronic liver inflammation through the activation of NLRP3 inflammasome.

Increased lysosomal membrane permeability and subsequent lysosomal rupture represent critical upstream signals that drive NLRP3 inflammasome activation, a regulatory mechanism also documented in AKI models. In the setting of ischemia-reperfusion (I/R)-induced AKI, hydroxychloroquine (HCQ) has been shown to attenuate renal injury by suppressing the activity of cathepsins (including CTSB, CTSD, and CTSL) as well as their redistribution from lysosomes to the cytosol. Of note, CTSB and CTSL—but not CTSD—have been demonstrated to directly participate in I/R-triggered NLRP3 inflammasome activation, suggesting that specific cathepsins serve as “danger signal” molecules released upon lysosomal disruption and exert selective roles in activating inflammatory responses (45).

#### 3.1.3 Role of mitochondrial dysfunction in NLRP3 inflammasome activation

Numerous studies have demonstrated that mitochondrial dysfunction assumes a crucial regulatory role in the activation of the NLRP3 inflammasome (46). Stress factors, including apoptosis, diminished autophagy activity, and NLRP3 inflammasome activators, can induce mitochondrial damage, leading to increased production of mitochondrial reactive oxygen species (mtROS). mtROS can further oxidize mtDNA, which is released into the cytoplasm and binds to the NLRP3 inflammasome to trigger its activation.

When mitochondrial dysfunction takes place, electron leakage occurs in complexes I and III of the electron transport chain, which results in the burst of mtROS. As a core triggering signal, mtROS can directly oxidatively modify the LRR domain of NLRP3. This process breaks the intramolecular disulfide bond, relieve its autoinhibitory conformation, and promotes NLRP3 oligomerization (47). Simultaneously, mtROS can mediate the dissociation of thioredoxin-interacting protein (TXNIP) from thioredoxin. The free TXNIP directly binds to NLRP3 to further stabilize the NLRP3 inflammasome assembly complex (48). Notably, linezolid, an oxazolidinone antibiotic, functions as aNLRP3 agonist, can activate the NLRP3 inflammasome independently of ROS. Both ROS-dependent and ROS-independent NLRP3 activation pathways lead to mitochondrial dysfunction, particularly in association with the characteristic mitochondrial lipid, cardiolipin. Cardiolipin can directly bind to NLRP3, and interfering with cardiolipin synthesis specifically inhibits NLRP3 inflammasome activation. mtROS oxidize cardiolipin in the inner mitochondrial membrane, disrupt the integrity of mitochondrial membrane, and induce the opening of the mitochondrial permeability transition pore (mPTP), providing a channel for the release of mtDNA (49).

mtDNA released into the cytosol has the potential to activate the cGAS-STING pathway, thereby inducing inflammation. When mitochondrial DNA undergoes oxidized (Ox-mtDNA), it binds to cytosolic NLRP3 in the cytoplasm, consequently triggering inflammasome activation. A recent study showed that Ox-mtDNA fragments, dependent on the mitochondrial permeability transition pore and VDAC channels, initiate the STING signaling pathway and activate NLRP3 inflammasome in the cytosol. In 2026, Liu et al. demonstrated that nucleotide metabolic reprogramming in obesity can result in mitochondrial dysfunction and drives the over - activation of NLRP3. Specifically, the inactivation of SAMHD1leads to the accumulation of cytoplasmic dNTPs, which enter mitochondria via PNC1/2, causing abnormal mtDNA regeneration. The newly generated mtDNA is prone to oxidation, forming ox-mtDNA. The excessive synthesis of mtDNA exacerbates mitochondrial damage, promotes the release of ox-mtDNA, and binds to the NACHT domain of NLRP3, ultimately mediating the hypersensitization activation of NLRP3 (50).

Mitochondrial dysfunction represents another critical upstream event driving NLRP3 inflammasome activation and has been established as a core pathogenic factor in various AKI models. In a contrast-induced AKI model, Lin et al. demonstrated, using both contrast agent-induced AKI mouse models and iohexol-stimulated HK-2 renal tubular epithelial cells, that NLRP3 inflammasome inhibition upregulates HIF1A expression and activates BNIP3-mediated mitophagy, thereby alleviating tubular epithelial cell apoptosis; conversely, BNIP3 deficiency abolished these renoprotective effects (51). This study indicates that NLRP3 negatively regulates the HIF1A/BNIP3 mitophagic pathway in the pathogenesis of contrast-induced AKI. In renal ischemia-reperfusion AKI models, ischemia/hypoxia triggers energy depletion, and the reperfusion phase precipitates oxidative stress, resulting in mitochondrial swelling, fragmentation, and membrane potential dissipation in tubular epithelial cells. Damaged mitochondria release substantial amounts of mtROS, which act as DAMPs to promote NLRP3 inflammasome assembly and activation, thereby instigating tubular inflammation and cell death. Zhu et al. demonstrated that enhancing PINK1/Parkin pathway-mediated mitophagy facilitates the timely clearance of damaged mitochondria, reduces mtROS accumulation, effectively curbs NLRP3 hyperactivation, and alleviates ischemia-reperfusion-induced renal injury (52). Collectively, these findings underscore that mitochondrial homeostasis disruption-driven NLRP3 inflammasome activation constitutes a shared pathogenic mechanism across diverse forms of AKI, rendering the targeting of mitochondrial homeostasis and the NLRP3 axis a promising universal therapeutic strategy for AKI.

### 3.2 Non-canonical and alternative activation pathways of NLRP3

**Figure 3:**
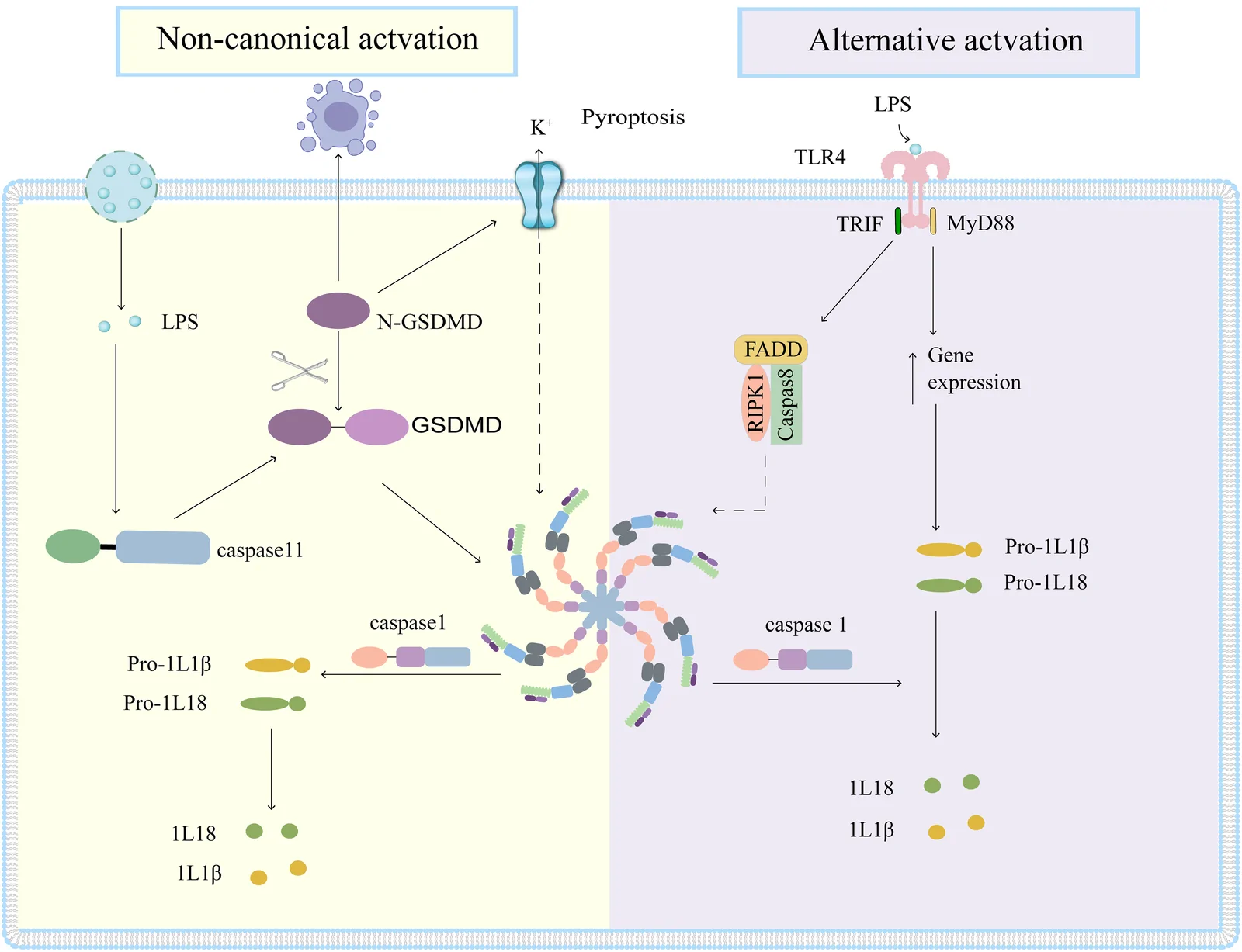
Non-canonical and alternative activation pathways of the NLRP3 inflammasome. Non-canonical activation of the NLRP3 inflammasome is initiated through cytosolic LPS detected by NF-κB-transcribed caspase-4/5/11, which cleave GSDMD to induce pyroptosis and K^+^ efflux. Concurrently, this process promotes the assembly of the NLRP3 inflammasome, activates caspase-1 and catalyzes the maturation of pro-IL-18 and pro-IL-1β, resulting in inflammatory responses. Alternative activation of the NLRP3 inflammasome depends on a single signal, takes place in monocytes, and is activated via TLR ligands through the TLR4-TRIF-RIPK1-FADD-caspase-8 signaling pathway, thereby promoting the release of IL-1β.

In addition to the canonical pathway, the NLRP3 inflammasome also possesses non-canonical and alternative activation pathways (**Figure 3**). The non-canonical activation pathway is primarily mediated by LPS on the surface of Gram-negative bacteria. LPS can directly enter the cytoplasm of host cell via endocytosis or transfection (53) and directly activate mouse caspase-11 (human homologs caspase-4/5), which is transcribed by NF-κB Activated caspase-11 subsequently cleaves GSDMD. GSDMD belongs to the gasdermin protein family, has an N-terminal fragment with pore-forming activity and a C-terminal fragment with autoinhibitory function. The N-terminal fragment of GSDMD forms pores on the cell membrane, leading to K^+^ efflux, inducing pyroptosis and subsequently activating the NLRP3 inflammasome, promoting IL-1β and IL-18 release (54, 55). The molecular mechanism by which NLRP3 is activated following caspase-11 activation has not been fully elucidated. A recent study identified the orphan nuclear receptor Nur77 as a key regulatory molecule of this pathway. Nur77 functions as a cytosolic LPS sensor, directly binding to LPS through its C-terminal domain and forming a complex with dsDNA released from the mitochondrial membrane after the cleavage of GSDMD by caspase-11. Conformationally changed Nur77 further interacts with NLRP3 and promotes its oligomerization, ultimately mediating caspase-1 activation and the maturation and release of IL-1β. Deficiency of Nur77 can significantly inhibit the non-canonical activation of NLRP3 in macrophages and alleviate the inflammatory response and organ damage of mice to endotoxin. This study, for the first time, identified the core mediating role of Nur77 downstream of caspase-11 and upstream of NLRP3 and refined the molecular regulatory network of NLRP3 non-canonical activation triggered by cytosolic LPS (56).

In addition to the canonical caspase-1-dependent NLRP3 inflammasome pathway, the caspase-11-mediated non-canonical activation pathway represents another important regulatory axis in AKI. In ischemia-reperfusion (I/R)-induced AKI models, activated caspase-11 amplifies tubular inflammation and cellular damage through two independent mechanisms. On one hand, Yin et al., using a murine renal I/R injury model established by bilateral renal pedicle clamping, demonstrated that injurious stimuli significantly upregulate caspase-11 expression in tubular epithelial cells. Activated caspase-11 cleaves the channel protein Panx1, promoting massive ATP release from cells; ATP subsequently acts on membrane-bound P2X7 receptors to trigger K^+^ efflux, which initiates NLRP3 inflammasome assembly and activation, culminating in the maturation and release of IL-1β. Notably, in caspase-11-deficient mice, I/R-induced Panx1 cleavage, NLRP3 inflammasome activation, as well as renal functional deterioration and tubular morphological damage were all markedly alleviated (57). On the other hand, caspase-11 can directly cleave GSDMD to generate the N-terminal pore-forming fragment, thereby initiating pyroptosis in tubular epithelial cells; disulfiram, an alcohol-aversion drug, has been shown to target the caspase-11/GSDMD axis and effectively mitigate I/R-mediated renal injury (58). Collectively, these findings in AKI models unequivocally establish that caspase-11, as a core molecule of the non-canonical pathway, not only acts as an upstream signal to promote NLRP3 inflammasome activation but also directly initiates pyroptotic cell death independent of NLRP3, jointly driving the progression of AKI.

The alternative activation pathway is independent of the classical “two-signal” and non-canonical caspase-11/4/5-dependent pathways. It is predominantly present in human primary monocytes and can be activated by a single signal from TLR ligands (e.g., LPS). However, it lacks the typical characteristics of NLRP3 inflammasome activation (such as K^+^ efflux, ASC speck formation, and pyroptosis). Instead, it is activated via the TLR4-TRIF-RIPK1-FADD-caspase-8 signaling axis, which subsequently promotes the release of IL-1β (59). Research has indicated that in mouse bone marrow-derived macrophages, stimulation of TLR4, TLR7, and TLR9 receptors can rapidly activate the NLRP3 inflammasome but only promotes IL-18 release, without inducing pyroptosis or IL-1β release (60). Sarah et al. demonstrated in primary human monocytes that various TLR ligands (including TLR1/2, TLR2/6, TLR4, and TLR7/8) can trigger the alternative activation of NLRP3 inflammasome through a single signal. This activation depends on the scaffolding function of NLRP3, caspase-1, and RIPK1 rather than their kinase activity. Independent of classical K^+^ efflux and pyroptosis, low-level of GSDMD channels are required to mediate the non - pyroptotic release of IL-1β, which contradicts the previous understanding that alternative activation was solely driven by the TLR4-TRIF axis (61).

To date, existing literature that directly investigates the association between the alternative activation pathway of the NLRP3 inflammasome and acute kidney injury remains relatively scarce. Nevertheless, mechanistic evidence strongly suggests a significant association. First, TLR4 serves as a key receptor mediating the alternative activation pathway; in acute kidney injury, factors such as ischemia-reperfusion injury and endotoxemia can significantly upregulate TLR4 expression in renal tissues, providing an upstream signaling foundation for this pathway. Second, Caspase-8, a critical effector molecule in the alternative pathway, plays an important role in tubular epithelial cell apoptosis, and its activation may directly contribute to tubular damage. Furthermore, inhibitors targeting specific molecular components of the alternative pathway—such as RIPK1, FADD, and Caspase-8—could offer more precise therapeutic strategies for acute kidney injury.

### 3.3 Regulatory mechanisms of NLRP3 activation

**Figure 4:**
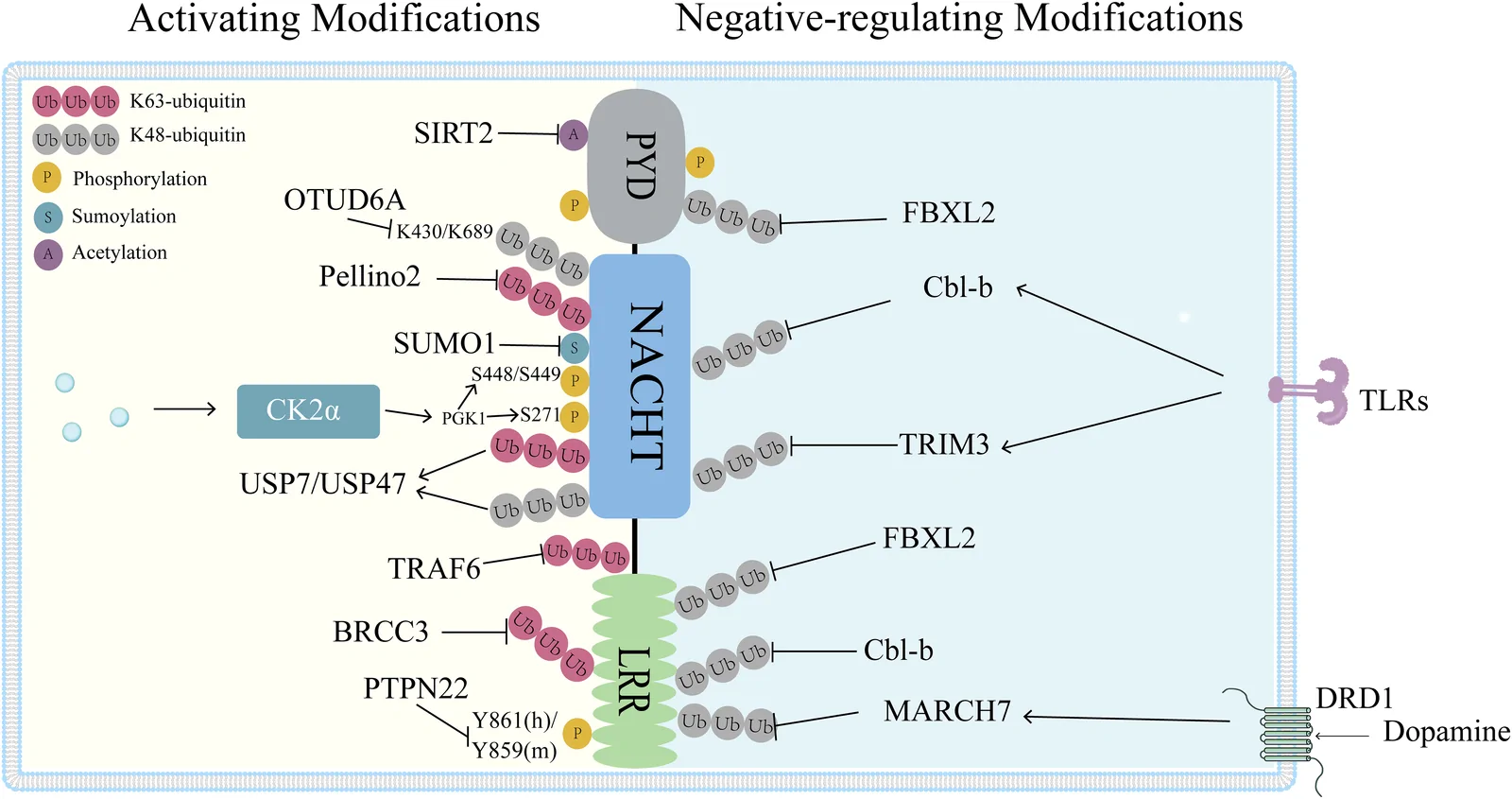
Post-translational modifications (PTM) of the NLRP3 inflammasome. NLRP3 is regulated by ubiquitination (Ub), phosphorylation (P), acetylation (A) and SUMOylation (S). The left-hand side depicts activating regulatory modifications, whereas the right-hand side illustrates inhibitory modifications that inhibit NLRP3 activation.

The activation signals of the NLRP3 inflammasome are finely regulated by a variety of PTMs, among which ubiquitination and phosphorylation represent the two most representative regulatory mechanisms. Emerging modifications such as acetylation and SUMOylation further complement and refine this regulatory network. These four classes of modifications interact in a coordinated manner, collectively participating in the comprehensive regulation of NLRP3 inflammasome activation throughout its entire process. It is noteworthy that PTMs of NLRP3 exhibit marked differences across various etiologies of AKI and among different cell types. This etiology-dependent disparity in PTM modifications represents one of the core contents first elaborated in this review (**Figure 4**).

#### 3.3.1 Regulation of NLRP3 inflammasome activation by ubiquitination

Ubiquitination plays a critical role in regulating the function of the NLRP3 inflammasome through various linkage types. The equilibrium between ubiquitination and deubiquitination is essential for controlling inflammasome activation. In the resting state, the NLRP3 protein is typically ubiquitinated, whereas it becomes deubiquitinated state during the priming and activation phases (62).

The majority f of the currently identified E3 ubiquitin ligases that target NLRP3 negatively regulate inflammasome activation by modifying NLRP3. Examples include F-box and leucine-rich repeat protein 2 (FBXL2), tripartite motif protein 31 (TRIM31), and Casitas B-lineage lymphoma b (Cbl-b). These E3 ligases are capable of inducing K48-linked ubiquitination of NLRP3, enabling its degradation by the proteasome (63). Furthermore, the neurotransmitter dopamine inhibits NLRP3 inflammasome activation by activating the dopamine D1 receptor (DRD1) signaling pathway. This pathway negatively regulates the NLRP3 inflammasome via the second messenger cyclic AMP (cAMP). cAMP binds to NLRP3 and promotes its ubiquitination and degradation through the E3 ubiquitin ligase MARCH7 (64).

It is noteworthy that not all E3 ubiquitin ligases negatively regulate NLRP3 activation. Studies has demonstrated that the E3 ubiquitin ligase pellino homolog 2 (Pellino2) can facilitate the activation of the NLRP3 protein during the priming phase by catalyzing the K63-linked polyubiquitination of NLRP3, thereby positively regulating NLRP3 activation (65). The E3 ubiquitin ligase TRAF6 has also been reported to directly interact with NLRP3, catalyzing the addition of K63-linked ubiquitin chains, promoting signal transduction rather than protein degradation (66). Unlike some negative regulators of NLRP3, TRAF6 enhances NLRP3 activation by promoting the oligomerization and assembly of the inflammasome complex (67).

Deubiquitination also plays a crucial role in regulating NLRP3 activity. Deubiquitinating enzymes (DUBs) are a category of specialized proteases that enhance or inhibit inflammasome activation by eliminating specific types of ubiquitin modifications, thereby reversing the function of ubiquitin ligases (67). The BRCA1/BRCA2-containing complex subunit 3 (BRCC3) is a key deubiquitinase enzyme that can specifically cleave the K63-linked polyubiquitin chains from NLRP3 and positively regulate the activation of the NLRP3 inflammasome (68). The deubiquitinases USP7 (Ubiquitin Carboxyl-Terminal Hydrolase 7) and USP47 enable NLRP3 to effectively interact with the adaptor protein ASC and pro-caspase-1, thereby promoting inflammasome assembly (33). They serve as positive regulators of NLRP3 inflammasome activation, regulate NLRP3 stability and activity through deubiquitination and facilitate its intracellular accumulation (69). The deubiquitinase OTUD6A directly binds to the NACHT domain of NLRP3 and specifically cleaves K48-linked polyubiquitin chain from the K430 and K689 sites of NLRP3, which enhances NLRP3 protein stability and results in an increase in inflammation and the IL-1β levels (70).

Studies have demonstrated that promoting NLRP3 protein degradation through ubiquitination modification represents an important protective mechanism for alleviating renal tubular injury in AKI. In sepsis-induced AKI (S-AKI), tissue inhibitor of metalloproteinases 2 (TIMP2) has been shown to exert renoprotective effects. Mechanistically, TIMP2 increases intracellular cyclic adenosine monophosphate (cAMP) levels, recruits the E3 ligase MARCH7, and promotes NLRP3 ubiquitination, directing it toward autophagic-lysosomal degradation, thereby effectively suppressing downstream pyroptotic pathways (including caspase-1 and GSDMD activation) and attenuating tubular cell injury. This finding not only reveals a novel function of TIMP2 as a biomarker but also suggests that exogenous TIMP2 administration or cAMP elevation could serve as a potential therapeutic strategy for S-AKI (71). The E3 ubiquitin ligase CHIP also alleviates pyroptosis in septic kidney injury through ubiquitination of NLRP3. CHIP indirectly associates with the LRR domain of NLRP3 via HSP70 and catalyzes K48-linked ubiquitination of NLRP3, marking it for proteasomal degradation, thereby inhibiting NLRP3/ASC inflammasome activation. This suggests that CHIP represents a potential therapeutic target for S-AKI (72). Conversely, ubiquitination has also been reported to indirectly promote NLRP3 inflammasome assembly and activation, thereby exacerbating AKI. In diabetic renal ischemia-reperfusion injury, the histone methyltransferase G9a is markedly upregulated. G9a promotes methylation of the transcription factor FoxO3a at lysine 262, which in turn facilitates its recognition by the E3 ligase TRIM21 and subsequent K48-linked polyubiquitination-mediated degradation at lysine 176. The degradation of FoxO3a leads to the loss of its anti-oxidative stress and anti-apoptotic functions, thereby promoting oxidative stress, NLRP3 inflammasome activation, and pyroptosis. Thus, the G9a-TRIM21-FoxO3a axis indirectly potentiates NLRP3 activity through ubiquitination-mediated degradation of a protective protein (73). TXNIP (thioredoxin-interacting protein), as an upstream activator of NLRP3, is itself subject to ubiquitination-mediated stability regulation. In radiation-induced AKI models, the transcription factor FOXA1 suppresses the transcription of the E3 ligase ITCH, resulting in TXNIP accumulation due to impaired ubiquitination-dependent degradation, which in turn exacerbates oxidative stress and NLRP3 inflammasome activation. Collectively, these findings indicate that targeting the ubiquitination networks of NLRP3 or its regulatory proteins (such as TXNIP) constitutes a critical node for therapeutic intervention in AKI (74).

Overall, ubiquitination and deubiquitination constitute a core regulatory system for NLRP3 inflammasome activation through dynamic and reversible post-translational modifications. These two processes act in mutual antagonism yet coordinated cooperation, collectively influencing multiple aspects of NLRP3 biology, including protein stability, degradation, inflammasome assembly, and catalytic activity. In the pathological microenvironment of AKI, the regulatory mechanisms governing NLRP3 ubiquitination have been relatively extensively elucidated in sepsis-associated AKI; however, comprehensive and in-depth comparative studies and mechanistic validation regarding its roles and specific regulatory patterns in other AKI subtypes remain lacking. Therefore, future investigations should further focus on the differential regulatory mechanisms of NLRP3 ubiquitination across distinct AKI subtypes, with the aim of providing novel therapeutic strategies for various forms of acute kidney injury.

#### 3.3.2 Regulation of NLRP3 inflammasome activation by phosphorylation

Phosphorylation can take place on the NACHT, LRR, and PYD domains of NLRP3 and is another crucial post-translational modification process, significant for NLRP3 inflammasome activation and assembly (75). The phosphorylation status of the LRR domain of NLRP3 can regulate the inflammasome. For instance, PTPN22-mediated dephosphorylation at residue Y859 (corresponding to Y861 in human NLRP3) can facilitate its activation (76). A recent study showed that PGK1 directly regulates NLRP3 inflammasome activation independently of its glycolytic function, demonstrating that CK2α-mediated phosphorylation of PGK1 at S271 promotes its binding to NLRP3 and induces subsequent phosphorylation of NLRP3 at S448/449, leading to inflammasome activation in macrophages pre - treated with LPS) (77). With the discovery of NLRP3 phosphorylation modification sites and their functions, the molecular regulatory mechanisms of its activation will be further clarified.

Regarding the phosphorylation modifications of the NLRP3 inflammasome in AKI, existing studies have primarily focused on two major regulatory aspects: the “priming signal” that governs its activation (e.g., NF-κB p65 phosphorylation) and the “assembly signal” (e.g., upstream kinase phosphorylation mediated by mitochondrial ROS). In sepsis-associated AKI, the phosphorylation regulation of the NLRP3 inflammasome involves multiple upstream signaling pathways. For instance, macrophage migration inhibitory factor (MIF) promotes phosphorylation of the NF-κB p65 subunit, thereby transcriptionally upregulating the renal NLRP3 inflammasome, amplifying tubular epithelial pyroptosis, and exacerbating septic AKI. These findings have been validated in both cecal ligation and puncture (CLP) mouse models and LPS-stimulated HK-2 cells (78). In renal ischemia-reperfusion injury, phosphorylation modification likewise serves as a critical determinant of NLRP3 inflammasome activation. Acidification of the tubular microenvironment following ischemia activates acid-sensing ion channel 1a (ASIC1a), which promotes NF-κB p65 phosphorylation, subsequently initiating NLRP3 and pro-IL-1β transcription, and ultimately aggravating renal I/R injury (79). Conversely, the natural plant-derived bioactive compound sarsasapogenin attenuates IRI by inhibiting both NF-κB p65 phosphorylation and the expression of NLRP3 inflammasome components (NLRP3, cleaved caspase-1, GSDMD-N), positioning it as a potential natural intervention agent for ischemic AKI. Klotho protein also exerts renoprotective effects by suppressing NF-κB phosphorylation (p-NF-κB), thereby reducing the expression of downstream pyroptosis-related proteins including NLRP3, caspase-1, and GSDMD (80). Mitochondrial oxidative phosphorylation (OXPHOS) dyshomeostasis and mtROS accumulation constitute important upstream triggers driving NLRP3 inflammasome activation. A study on spermidine reported that the natural polyamine spermidine restores mitochondrial respiratory function in macrophages, enhances OXPHOS levels, and alleviates mtROS overload, thereby blocking NLRP3 inflammasome activation and subsequent release of pro-inflammatory cytokines, thus conferring renoprotection (81). Notably, this metabolic regulatory effect is dependent on the eIF5A hypusination pathway, rather than directly intervening in NLRP3 phosphorylation modifications. In contrast-induced nephropathy, Drp1-mediated mitochondrial fission represents a critical pathogenic event. Phosphorylation of Drp1 at Ser616 is upregulated, leading to mitochondrial dynamic imbalance, which in turn promotes mtROS production and ultimately activates the inflammasome via the TXNIP-NLRP3 axis. Inhibition of Drp1 phosphorylation (Ser616) effectively downregulates NLRP3 expression (82).

Notably, current literature still lacks investigations on the direct phosphorylation sites and functional roles of the NLRP3 protein itself across different types of AKI; instead, the vast majority of investigations have concentrated on phosphorylation modifications of upstream regulatory molecules that govern NLRP3 activation. Elucidating the direct phosphorylation sites of the NLRP3 protein, and delineating how such modifications modulate its interaction with ASC and caspase-1 as well as inflammasome assembly, therefore represents a critical knowledge gap warranting future investigation. Research in this field can offer novel strategies for the targeted therapy of AKI, particularly for the development of inhibitors or modulators that target specific phosphorylation sites on NLRP3.

#### 3.3.3 Regulation of NLRP3 inflammasome activation by other PTMs

Acetylation modification also plays an important role in the full activation of the NLRP3 inflammasome. Studies have demonstrated (83) that, upon stimulation with the second signal for NLRP3 inflammasome assembly, the histone acetyltransferase KAT5 serves as the acetyltransferase mediating NLRP3 acetylation, catalyzing acetylation of NLRP3 at lysine 24 (K24), which ultimately promotes NLRP3 activation. This modification has also been confirmed in a cisplatin-induced AKI (CI-AKI) model, representing a critical step for NLRP3 inflammasome assembly and activation. Astragaloside IV (AS-IV), a major bioactive component of *Astragalus membranaceus*, has been shown to bind KAT5 and suppress both its expression and catalytic activity, thereby blocking KAT5-mediated NLRP3 acetylation at K24, ultimately inhibiting NLRP3 inflammasome-mediated pyroptosis and alleviating renal injury. This provides direct evidence for targeting NLRP3 acetylation to ameliorate cisplatin nephrotoxicity (84). In a S-AKI model, Zn²^+^ was found to upregulate Parkin acetylation levels by inhibiting SIRT7 activity. Although this study primarily focused on the regulation of mitophagy by Parkin acetylation, the findings clearly demonstrated that Zn²^+^, through this mechanism, ultimately suppressed NLRP3 inflammasome activation and pyroptosis, thereby improving SI-AKI (85).

SUMOylation also represents an important post-translational modification process regulating NLRP3 function. The E3 SUMO ligase tripartite motif-containing protein 28 (TRIM28) serves as a key regulatory factor in mediating NLRP3 SUMOylation, which enhances NLRP3 inflammasome activation by upregulating NLRP3 expression. TRIM28 binds to NLRP3 and promotes its modification by SUMO1, SUMO2, and SUMO3, thereby inhibiting NLRP3 ubiquitination and proteasomal degradation (86). SUMO1-mediated SUMOylation of NLRP3 at Lys204 facilitates ASC oligomerization and NLRP3 inflammasome activation (87). However, other studies have demonstrated that SUMOylation of NLRP3 mediated by the E3 SUMO ligase MAPL restricts NLRP3 inflammasome activation, suggesting that multi-site SUMO conjugation of NLRP3 serves as an important negative regulatory mechanism in innate immune signaling (88). Nevertheless, current evidence regarding SUMOylation modifications in various types of AKI remains scarce, representing a knowledge gap in the mechanistic study of kidney diseases.

Palmitoylation is a reversible lipid post-translational modification in which palmitoyl acyltransferases (ZDHHC family) covalently conjugate palmitic acid via thioester bonds to cysteine residues of target proteins, thereby regulating protein subcellular localization, membrane anchoring stability, and protein-protein interactions. Depalmitoylating enzymes (APT and ABHD families) remove lipid chains to achieve dynamic and reversible regulation, representing a unique membrane-localization regulatory pathway for NLRP3 that is distinct from phosphorylation and ubiquitination (89). Studies have shown that multiple cysteine sites on NLRP3 can undergo palmitoylation, with these modifications exerting divergent effects on inflammasome activation. ZDHHC5 promotes NLRP3-NEK7 interaction and inflammasome assembly by catalyzing palmitoylation of the NLRP3 LRR domain (90). ZDHHC7 specifically catalyzes palmitoylation of Cys126 within the NLRP3 PYD domain, which is essential for NLRP3 localization to the trans-Golgi network (TGN) and subsequent inflammasome activation, representing the most mechanistically well-characterized palmitoylation pathway to date (91). Additionally, ZDHHC17 catalyzes palmitoylation at Cys419 of NLRP3, facilitating the formation of the NLRP3-NEK7 complex (92). In contrast, ZDHHC12-mediated palmitoylation at human NLRP3 Cys844 promotes its degradation via chaperone-mediated autophagy, thereby constraining excessive inflammasome activation (93). This dual regulatory mechanism reflects the exquisite control of NLRP3 activity by the cell. In AKI, mitochondrial dysfunction induced by ischemia-reperfusion injury or nephrotoxic agents serves as a critical trigger for NLRP3 activation. Studies have demonstrated that ZDHHC5 activates the inflammasome by enhancing NLRP3 palmitoylation, which in turn exacerbates mitochondrial damage, suggesting that palmitoylation may play a pivotal role in the mitochondrial damage-inflammation vicious cycle in AKI (94). AKI is frequently accompanied by lipid metabolic dysregulation, and palmitoylation precisely links metabolism and inflammation. LPS-induced metabolic reprogramming leads to palmitic acid accumulation in cells, which promotes palmitoylation of NLRP3 at Cys126 and Cys898, enhancing its localization to and activation at the TGN membrane. This immunometabolic crosstalk may represent an important mechanism contributing to aggravated tubular epithelial injury in AKI (95). Currently, research on NLRP3 palmitoylation in the AKI field remains incomplete. The diverse ZDHHC family enzymes that regulate NLRP3 subcellular localization, protein stability, and activation levels offer new perspectives for elucidating the mechanisms of inflammatory dysregulation in AKI and provide potential avenues for targeted intervention. Future systematic investigations integrating AKI animal models and clinical specimens will help clarify the specific roles of palmitoylation in AKI and facilitate the translational application of related therapeutic strategies.

In summary, the activity status of NLRP3 at different stages is coordinately determined by multiple classes of PTMs in a temporally defined manner. Ubiquitination primarily regulates NLRP3 protein stability, phosphorylation and acetylation mainly mediate signal transduction and transcriptional initiation, while palmitoylation governs membrane localization and inflammasome complex assembly. This temporal regulatory network may exhibit differential activation patterns across distinct etiologies of AKI; however, direct comparative evidence remains insufficient. Notably, ubiquitination is currently the only PTM pathway that has been validated to exert direct renoprotective effects across multiple AKI subtypes. In contrast, intervention evidence for phosphorylation modifications is primarily confined to the upstream NF-κB pathway; acetylation modification has only been directly demonstrated in cisplatin-induced AKI; and direct experimental evidence for palmitoylation and SUMOylation modifications in AKI renal tissues remains incomplete or extremely limited. Therefore, current research on PTM regulatory mechanisms of NLRP3 activation in the AKI field faces the following critical gaps that urgently need to be addressed: (1) insufficient identification of direct PTM sites on the NLRP3 protein, particularly the severe lack of direct validation of phosphorylation and SUMOylation sites across various cell types in renal tissues; (2) the absence of systematic comparative analyses of PTM modification profiles among different AKI subtypes, with currently somewhat more evidence available from sepsis-associated AKI, whereas studies on PTMs in ischemic and contrast-induced AKI remain relatively weak; and (3) the complete lack of translational validation of PTM-targeted drugs in large animal models of AKI and clinical trials.

## 4 Role of the NLRP3 inflammasome in different types of AKI

**Figure 5:**
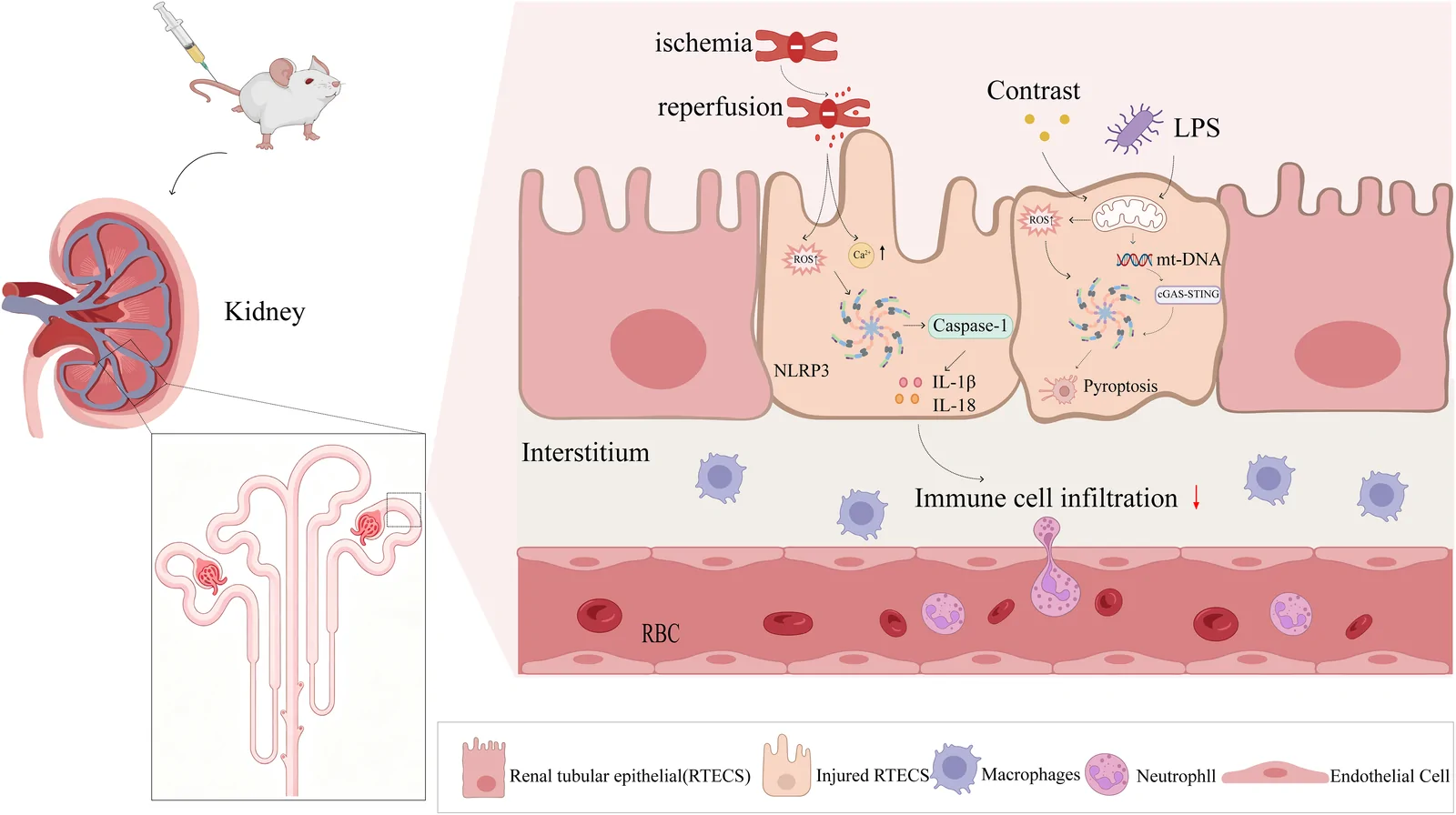
The role of the NLRP3 inflammasome in three types of AKI. Upon exposure to injury factors such as ischemia-reperfusion, contrast agents, or lipopolysaccharide (LPS) stimulation, renal tubular epithelial cells (RTECs) sustain damage, resulting in an elevated intracellular Ca²^+^ concentration. This elevated concentration activates the NLRP3 inflammasome, which subsequently activates caspase-1 and promotes the maturation and release of pro-inflammatory cytokines, such as IL-1β and IL-18. Injured RTECs release inflammatory signals that induce infiltration of immune cells, including macrophages and neutrophils, into the renal interstitium, further amplifying local inflammation and exacerbating tubular injury and renal dysfunction.

The NLRP3 inflammasome plays a crucial pathogenic role in AKI induced by various etiologies. Owing to the disparities in the pathophysiological mechanisms of different causes, the activation mode, effector cells, and downstream signaling pathways of the NLRP3 inflammasome also exhibit distinct characteristics. The subsequent section elaborates on the research progress in ischemia-reperfusion AKI, sepsis- induced AKI, and contrast- induced AKI (**Figure 5**).

### 4.1 Ischemia-reperfusion AKI

Ischemia-reperfusion injury (IRI) refers to the pathophysiological state where the blood supply to an organs is initial restricted, resulting in tissue ischemia and hypoxia. Subsequently, the restoration of blood perfusion and reoxygenation occurs, which further exacerbates tissue damage and triggers a severe inflammatory response (96). Ischemia-reperfusion acute kidney injury (IR-AKI) is one of the most prevalent types of AKI in clinical practice, and it is more frequently observed in renal surgery, kidney transplantation, cardiovascular surgery, severe shock and other clinical situations (97).

During the ischemic phase, inadequate renal blood perfusion gives rise to hypoxia and energy metabolism disorders, resulting in the accumulation of oxygen free radicals and reactive nitrogen species, thereby causing damage to renal tubular epithelial cells. Nevertheless, the rapid restoration of blood and oxygen supply during reperfusion results in a substantial production of ROS and intracellular Ca²^+^ overload. This, in turn, triggers the infiltration of immune cell and an excessive inflammatory response, ultimately leading to apoptosis, necrosis, and irreversible damage of renal tubular epithelial cells (98).

Studies have demonstrated that NLRP3 is highly expressed in renal tubular epithelial cells, and the depletion of NLRP3 can substantially attenuates renal ischemia-reperfusion injury (99), suggesting that NLRP3 directly participates in the pathogenesis of renal ischemia-reperfusion acute injury through its action on renal tubular epithelial cells. Wen et al. using a mouse renal IR model and a human renal tubular epithelial cell (HK-2) hypoxia-reoxygenation model, demonstrated that renal IR can result in mitochondrial damage and a substantial release of mROS in renal tubular epithelial cells, which promotes the dissociation of TXNIP from thioredoxin (Trx), allowing TXNIP to bind NLRP3 and initiate NLRP3 inflammasome assembly and activation (48). Subsequently, caspase-1 mediates the release of pro-inflammatory factor and pyroptosis, which leads to renal tubular necrosis, inflammatory infiltration of renal tissue, and the deterioration of renal function. The knockout of NLRP3 or silencing of TXNIP can significantly inhibit inflammasome activation and attenuated kidney injury, confirming that the mROS-TXNIP-NLRP3 axis is a key signaling pathway mediating IR-AKI, and TXNIP is the essential molecule for mROS to activate NLRP3 in this pathway. In addition, Pang et al. observed that the expression level of the pyroptosis executive protein GSDMD was significantly elevated in the IR-AKI mouse model, suggesting that NLRP3 inflammasome-mediated pyroptosis plays an important role in IR-AKI (100). A recent study found that IR can significantly activate the pyroptosis pathway in renal tissue and upregulate pyroptosis-related proteins such as NLRP3, GSDMD, and IL-1β, accompanied by an increase in serum creatinine/urea nitrogen and exacerbation of renal tubular pathological damage. Sodium aescinate (SA) exerted an anti-pyroptotic effect by inhibiting the AKT/NLRP3 signaling pathway (101). Inhibition of AKT phosphorylation blocked NLRP3 inflammasome activation and downstream pyroptotic.

Bakker et al. used the bone marrow chimeric mouse models to demonstrate that the expression of NLRP3 was significantly upregulated during the repair phase of renal (I/R. Moreover, the regulatory impacts of NLRP3 originating from leukocytes and renal intrinsic cells on renal injury and repair differed. The findings indicated that NLRP3 expression reached its peak during the I/R repair stage. NLRP3 from both cell types could exacerbated renal dysfunction post-injury, and this process was independent of the expression of IL-1β. Further mechanistic analysis revealed that leukocyte-derived NLRP3 exacerbated renal tubular epithelial cell apoptosis by diminishing macrophage infiltration, whereas renal tissue-derived NLRP3 significantly inhibited the proliferation of renal tubular epithelial cells and upregulated the expression of hepatocyte growth factor (HGF). *In vitro* experiments also confirmed that NLRP3 gene knockout enhanced the repair and structural reconstruction capacity of renal tubular epithelial cells. This study clarified the tissue-specific role of NLRP3 in renal I/R repair (102). Ning et al. validated the expression characteristics of TRIM44 in the injury models through the *in vivo* mouse IR models and the *in vitro* hypoxia/reoxygenation (H/R) model of renal tubular epithelial cells. Results showed that the up - regulation of TRIM44 expression effectively reduced the levels of serum creatinine and urea nitrogen in mice, alleviated pathological damage of renal tubular epithelial cell necrosis, exfoliation, and inflammatory infiltration of renal tissue. Simultaneously, it notably suppressed the expression of pyroptosis-related markers, including the secretion of cleaved caspase-1, GSDMD-N, IL-1β and IL-18. This research identified the regulatory role of the TRIM44-NLRP3-pyroptosis axis in IR-AKI (103).

Existing studies indicate that NLRP3 activation in IR-AKI involves at least two distinct pathways: the mtROS-TXNIP-NLRP3 canonical activation pathway mediates acute-phase inflammation and pyroptosis, whereas the sustained expression of NLRP3 during the repair phase may influence tissue remodeling in a cell source-dependent manner. In addition, the discovery of the TRIM44-NLRP3-pyroptosis axis suggests the existence of an endogenous negative feedback mechanism regulating NLRP3. The differential functions of NLRP3 derived from immune cells versus renal parenchymal cells, as revealed by bone marrow chimera experiments, indicate that the contributions of NLRP3 from these distinct cellular sources to renal injury and repair are divergent—a finding that holds important implications for the design of future targeted therapeutic strategies. However, current studies are heavily reliant on mouse models and lack temporal dynamic validation using clinical renal biopsy specimens, making it uncertain whether the mtROS-TXNIP pathway similarly serves as a core axis in human IR injury. Moreover, most existing investigations have focused on the whole-kidney tissues, and there is a lack of targeted intervention exploration of renal tubules and immune cell subsets. Therefore, more cell-specific gene interventions can be carried out in the future to distinguish the differential functions of NLRP3 in different cells during acute phase injury and repair phase remodeling.

### 4.2 Sepsis-induced AKI

Sepsis is a life-threatening multiple organ dysfunction syndrome caused by a dysregulated host immune response to infection, characterized by organ failure and various physiological and serological abnormalities (104). Sepsis is a prevalent complication among hospitalized and critically ill patients, and it can induce AKI through multiple mechanisms, including glomerular injury, inflammation response, microvascular dysfunction, and renal tubular injury.

In basic research, models of S-AKI are typically established through the intraperitoneal injection of LPS in mice. LPS participates in the pathogenesis of S-AKI by inducing oxidative stress and inflammation response. Li Z et al. (105) initially verified the high expression of NLRP3 in human kidney tissue of S-AKI patients and found that NLRP3 gene deficiency significantly attenuated S-AKI. Moreover, the study also revealed that ferroptosis participates in LPS-induced AKI, and NLRP3 deficiency alleviated this ferroptotic injury.

LPS has the capacity to activate the NLRP3 inflammasome by upregulating the expression of NLRP3, caspase-1, and IL-1β proteins in the injured renal tissue. NF-κB serves as a crucial upstream transcription factor for NLRP3 inflammasome activation. Shi J et al. demonstrated that the inhibition of the NF-κB pathway, followed by the suppression of NLRP3 inflammasome activation, effectively prevented LPS-induced AKI (106). In recent years, the significance of the mtDNA (mtDNA)-cGAS-STING axis in S-AKI has garnered substantial attention. Recent studies showed that mtDNA can initiate the cGAS-STING signaling axis, which participates in the onset and progression of S-AKI by activating the NLRP3 inflammasome. Mitigating mtDNA accumulation or suppressing the cGAS-STING axis can alleviate renal tubular injury (107).

At the molecular regulatory level, studies have confirmed that (108) LPS can induce the abnormally high expression of NLRP3 in HK-2 cells. Moreover, the inhibition of NLRP3 can significantly reduce the apoptosis of renal tubular epithelial cell and the activation of NLRP3. Mechanism studies revealed that lncRNA OIP5-AS1 targeted and adsorbed miR-186-5p through the competing endogenous RNA (ceRNA) mechanism, thereby relieving its inhibitory effect on NLRP3 and activating the TLR4/NF-κB pathway. Experiments on rat kidney tissue further verified the correlation among OIP5-AS1, miR-186-5p, and NLRP3 expression. Therefore, OIP5-AS1 can activate NLRP3 by regulating the miR-186-5p/NLRP3 axis, which aggravates renal inflammation and tissue damage. Using C57BL/6J mice subjected to intraperitoneal LPS injection to establish a S-AKI model, Wang et al. investigated the renoprotective mechanisms of electroacupuncture. They found that electroacupuncture intervention alleviated acute kidney injury in septic mice by inhibiting the activation of the P2RX7/NLRP3 signaling pathway, which subsequently reduced the release of downstream inflammatory cytokines IL-1β and IL-18 and attenuated pyroptosis in renal tubular epithelial cells, confirmed that the P2RX7/NLRP3 axis represents a key regulatory pathway through which electroacupuncture exerts its renoprotective effects in S-AKI (109). Lu J et al. used LPS to establish a S-AKI mouse model and an *in vitro* model of HK-2 cell. Through gene knockdown, overexpression, and inhibitor intervention experiments, they demonstrated that acetyl-CoA synthetase 2 (ACSS2) was abnormally highly expressed in septic kidney injury; ACSS2 could upregulate KLF5 and activates the NF-κB signaling pathway. This, in turn, promoted the activation of NLRP3 inflammasome, induced pyroptosis of renal tubular epithelial cells and massive release of a large number of inflammatory factors, indicated that the ACSS2/KLF5/NF-κB pathway is a crucial molecular mechanism for regulating inflammation and pyroptosis in S-AKI (110).

Collectively, NLRP3 activation in S-AKI involves multiple levels of regulation: at the transcriptional level, LPS upregulates the expression of NLRP3 components via the NF-κB pathway; at the mitochondrial level, mtDNA amplifies inflammatory signals through the cGAS-STING axis; and at the metabolic level, ACSS2 promotes NLRP3 activation via the KLF5/NF-κB pathway. Thus, NLRP3 activation in S-AKI is highly dependent on NF-κB-mediated transcriptional upregulation, suggesting that blockade of the NF-κB pathway may reduce total NLRP3 protein levels and achieve upstream control of inflammasome activation. Nevertheless, although multiple signaling pathways have been identified, the hierarchical relationships among them remain poorly defined. Future studies should prioritize the identification of predominant activation pathways corresponding to clinically common pathogens, and meanwhile establish renal biopsy cohort studies in infected patients to bridge the gap between animal experiments and clinical practice.

### 4.3 Contrast-induced AKI

Contrast-induced acute kidney injury (CI-AKI) is defined as a pathological condition of acute renal function impairment following the administration of contrast media, particularly iodine-based contrast agents (111). Currently, renal tubular injury resulting from renal hypoxia/ischemia is regarded as the primary pathogenesis of CI-AKI.

Shen et al. using HK-2 cells and a uninephrectomy model, found that the expression of NLRP3 and ASC was significantly increased after contrast agent treatment. The knockout of NLRP3 or ASC attenuated IL-1β and IL-18 secretion and inhibited the apoptosis of HK-2 cells, thereby confirming that NLRP3 mediates CI-AKI by regulating the apoptotic pathway (112). On the other hand, Lin et al. found that contrast agents can induce mitochondrial damage in renal tubular epithelial cells, leading to massive ROS generation, which specifically activates the PINK1-Parkin pathway and initiating mitophagy. Mitophagy can inhibit the activation of NLRP3 by eliminating damaged mitochondria, thereby exerting a renoprotective effect (113).

Notably, the regulatory effects of contrast agents on NLRP3 differ among various renal cell types. One study demonstrated that contrast agents are reabsorbed and concentrated by dipeptidase-1 (DPEP1) at the brush border of the renal tubular. This process facilitates the uptake of the contrast agent by renal interstitial macrophages, specifically activates NLRP3 in macrophages, and leads to the release of IL-1β, thereby causing kidney injury. Nevertheless, the direct injury to renal tubular epithelial cells by contrast agents is independent of NLRP3 (114). Li Y et al. established a CI-AKI cell model by exposing HK-2 cells to iohexol and demonstrated that the contrast agent induces massive accumulation of intracellular ROS, which in turn activates the NLRP3/Caspase-1/GSDMD signaling pathway and triggers pyroptosis in renal tubular epithelial cells. Notably, baicalin alleviates contrast agent-induced tubular epithelial cell injury by inhibiting ROS production and downregulating the expression of key pathway proteins including NLRP3, Caspase-1, and GSDMD (115). Song L et al. employed a rat model of CI-AKI *in vivo* and intervened with astaxanthin, demonstrating that astaxanthin exerts renoprotective effects against CI-AKI by inhibiting ROS-mediated NLRP3 inflammasome activation (116).

The aforementioned researches indicate that NLRP3 activation in CI-AKI involves cell type-dependent mechanisms. In renal tubular epithelial cells, NLRP3 primarily participates in the regulation of apoptosis and pyroptosis through the mitochondrial damage-ROS pathway, whereas NLRP3 activation in renal interstitial macrophages depends on DPEP1-mediated contrast agent uptake. Furthermore, PINK1-Parkin-mediated mitophagy serves as an endogenous protective mechanism, and the interventional effects of natural products such as baicalin and astaxanthin collectively underscore the central role of the ROS-NLRP3 pathway in CI-AKI. However, whether the differential effects of contrast agents on these two cell types are determined by intrinsic cellular differences or by disparities in intracellular contrast agent concentrations remains unclear, as no direct experimental evidence is currently available, warranting further investigation.

### 4.4 Common patterns and differential mechanisms of NLRP3 in different types of AKI

A comprehensive comparison of NLRP3 activation characteristics across the three AKI subtypes reveals the following patterns:

1. Common activation module: Mitochondrial dysfunction serves as a shared upstream event driving NLRP3 activation across all three AKI subtypes. In IR-AKI, mtROS activates NLRP3 through the TXNIP pathway; in S-AKI, mtDNA participates in inflammatory signal amplification via the cGAS-STING pathway; and in CI-AKI, contrast agents directly induce mitochondrial damage and massive ROS accumulation. These findings suggest that mitochondrial quality control represents a potential target for broad-spectrum intervention strategies.
2. Core differential dimensions: Available evidence indicates that the three AKI subtypes differ in the following aspects:Dominant activation signals—IR-AKI is predominantly driven by mtROS/TXNIP; S-AKI involves dual pathways of LPS/NF-κB and mtDNA/cGAS-STING; CI-AKI involves lysosomal damage and ROS. Effector cells—In IR-AKI, NLRP3 exerts a direct role in renal tubular epithelial cells; S-AKI involves both macrophages and tubular cells; CI-AKI exhibits a binary differentiation between macrophages and tubular cells. Expression dynamics—IR-AKI displays a secondary peak of NLRP3 expression during the repair phase; S-AKI exhibits sustained LPS-induced NLRP3 activation; CI-AKI shows acute self-limiting contrast-induced NLRP3 activation.
3. Etiological implications for therapeutic strategies: The above differences suggest that distinct AKI subtypes may require differentiated NLRP3-targeted strategies. In IR-AKI, the expression pattern of NLRP3 during the repair phase underscores the need to consider the timing of inhibition; in S-AKI, the presence of multiple upstream signals suggests that combined intervention may be necessary; in CI-AKI, cell type-specific differences highlight the importance of targeted delivery strategies. However, no parallel studies have yet directly compared the therapeutic efficacy of NLRP3 targeting across the three AKI subtypes, and the above implications await experimental validation.

## 5 Current status of targeting the NLRP3 inflammasome for AKI intervention

The NLRP3 inflammasome plays a critical role in the occurrence and progression of various types of AKI. Therefore, developing intervention strategies aimed at NLRP3-related signaling pathways is anticipated to emerge as a novel direction for AKI treatment. Currently, there exists various biological or chemical inhibitors targeting different components of the NLRP3 inflammasome (**Table 1**). However, the efficacy and safety of these inhibitors in the context of AKI require further verification. The following sections review research progress from the perspectives of targeting NLRP3 itself, regulating mitochondrial function, interfering with K^+^ channels, and targeting caspase-1.

**Table 1:** Current status of therapeutic approach targeting NLRP3 in acute kidney injury.

Drug name	Therapeutic target	Inhibition mechanism	Type of AKI	References
MCC950	NLRP3	Binds to the NACHT domain, thereby inhibiting ATPase activity and oligomerization.	IR-AKI	(117)
OLT1177	NLRP3	Inhibits NLRP3 oligomerization and ASC recruitment, blocks caspase-1 activation and release IL-1β/IL-18	CI-AKI	(120)
ginsenoside Rg3	NLRP3	Blocks IL-1β secretion and caspase-1 activation	S-AKI	(123)
oridonin	NLRP3	Blocks the interaction between NEK7 and NLRP3	IR-AKI	(124)
Micheliolide	mitochondria	Promote mitophagy, eliminate ROS	S-AKI	(125)
Berberine	mitochondria	Enhance mitophagy via AMPK	CI-AKI	(126)
β-hydroxybutyrate	K^+^channel	Inhibit K^+^ efflux and reduce ASC oligomerization and speck formation	Unknown	(127)
Glibenclamide	K^+^channel	ATP-sensitive K^+^ channel inhibitor	CKD	(129)
Rosmarinic Acid	Caspase-1	Block caspase-1 activation	CP-AKI	(130)
VX-740/VX-765	Caspase-1	Selectively inhibit caspase-1	Unknown	(131)
AC-YVAD-CMK	Caspase-1	Irreversible inhibitor of caspase-1	S-AKI	(132)
Baicalin	Caspase-1	Inhibit pyroptosis mediated by the ROS/ NLRP3/caspase1 /GSDMD pathway	CI-AKI	(115)
cordycepin	Caspase-1	Directly bind to and block the NLRP3/Caspase-1 /GSDMD pathway	S-AKI	(133)

### 5.1 Drugs targeting NLRP3

MCC950 (also known as CP-456773, CRID3) is currently the most extensively studied and most specific small-molecule inhibitor of NLRP3 (17). It is a synthetic small molecule sulfonylurea compound that can bind to the NACHT domain of NLRP3, and inhibit its ATPase activity and oligomerization, thus blocking the activation of NLRP3 inflammasome (117). Cornelius et al. (118) found in a sepsis model induced by cecal ligation and puncture in rats that the activation of NLRP3 could mediate sepsis-induced platelet activation, which subsequently triggered endothelial dysfunction and multi-organ damage. The targeted inhibition of NLRP3 by MCC950 significantly reduced the platelet activation rate in septic rats, effectively inhibited IL-1β/IL-18 releases and caspase-1 activation, and alleviated pathological damage to the kidney and lung tissue. Zou et al. (119) further verified that MCC950 reduced acute graft injury after kidney transplantation in rats via the NLRP3- IL-1β-chemokine axis by inhibiting the NLRP3 inflammasome, which for the first time revealed that the inhibition of NLRP3 could alleviate acute allograft rejection triggered by long-term ischemia. This study offers a crucial experimental basis for the intervention of IRI in renal transplantation.

OLT1177 (Dapansutrile) is an orally administrable β-sulfonyl nitrile molecule that specifically inhibits NLRP3 oligomerization and ASC recruitment, blocks caspase-1 activation, and prevents the maturation and release of IL-1β/IL-18. Furthermore, it exerts no significant influence on other inflammasomes such as NLRC4 and AIM2 (120). Tan et al. (121) demonstrated that contrast agents can induce the release of S100A8/A9, which activates the NLRP3 inflammasome by binding to TLR4. OLT1177 can impede the crucial link in this pathway, reducing renal tubular inflammation and apoptosis, and protecting renal function. This provides a crucial theoretical basis for targeting NLRP3 (e.g., with OLT1177) in the treatment of CI-AKI. Elsayed et al. (122) further discovered that OLT1177 interrupted the inflammasome activation cascade by targeting the NACHT domain of NLRP3 and notably decreased the levels of serum creatinine (SCr), blood urea nitrogen (BUN), and kidney injury molecule-1 (KIM-1) in a folic acid (FA)-induced AKI mouse model., significantly reduces This study reverses the impairment of renal function caused by FA and enhances renal function, which presents a new strategy for targeted therapy of drug-induced AKI and the transition from AKI to CKD.

Additionally, certain natural drugs exhibit direct inhibit NLRP3 activity. For instance, ginsenoside Rg3 blocks IL-1β secretion and caspase-1 activation by inhibiting LPS-induced priming signals and NLRP3 inflammasome activation in human and mouse macrophages (123). Oridonin prevents the assembly and activation of NLRP3 inflammasome by blocking the interaction between NEK7 and NLRP3 interaction (124), both represent natural inhibitors directly targeting NLRP3.

### 5.2 Drugs targeting mitochondria

Mitochondrial dysfunction represents the crucial upstream event of NLRP3 inflammasome activation. Therefore, improving mitochondrial quality and promoting mitophagy serve as an related strategies to inhibit NLRP3 activation.

Micheliolide (MCL) is a natural sesquiterpene lactone compound extracted from plants of the Magnoliaceae family, possessing anti-inflammatory, antioxidant, and anti-pyroptotic effects without obvious toxicity. Lei et al. demonstrated that Micheliolide (MCL) can promote mitophagy by activating the Nrf2/PINK1/Parkin axis, thereby clearing damaged mitochondria and mitochondrial ROS, suppressing NLRP3 inflammasome activation, and improving LPS-induced septic AKI (125). This study provides new mechanistic insights and potential therapeutic candidates for S-AKI.

Berberine (BBR) is a type of benzylisoquinoline quaternary ammonium alkaloid. Studies have indicated that BBR inhibits NLRP3 inflammasome activation through AMPK-dependent enhancement of mitophagy, exerting a protective effect against contrast-induced acute kidney injury, offering a rapidly translatable natural drug strategy for the prevention of CI-AKI (126). However, it should be noted that the protective effect of this drug has only been validated in mouse model of CI-AKI, and there is still a dearth of evidence from large animal and human clinical trials.

### 5.3 Drugs targeting K^+^ channels

K^+^ efflux is one of the key upstream signals for NLRP3 inflammasome activation, therefore, interfering with K^+^ channels or stabilizing intracellular K^+^ concentration represents another potential strategy to inhibit NLRP3 activation.

β-hydroxybutyrate (BHB) is an endogenous ketone body, has been identified as an inhibitor of the NLRP3 inflammasome in recent years. BHB is capable of inhibiting ASC oligomerization and speck formation and preventing K^+^ efflux (127). Studies have shown that BHB can transform the phenotype of macrophage from a pro-inflammatory (M1) to an anti-inflammatory (M2) profile, implying that BHB might be a potential drug for treating AKI-related diseases by inhibiting the NLRP3 inflammasome (128). However, direct evidence regarding its role in AKI models still needs further accumulation.

Glibenclamide is a second-generation sulfonylurea drug widely used clinically for type 2 diabetes mellitus. Lamkanfi et al. (129) found that glibenclamide was capable of binding to ATP-sensitive K^+^ channels, thereby inhibiting NLRP3 inflammasome activation. As an NLRP3 inflammasome inhibitor, glibenclamide effectively inhibits adenine-induced CKD and renal interstitial fibrosis in rats.

### 5.4 Drugs targeting caspase-1

Caspase-1 is a downstream effector molecule of the NLRP3 inflammasome. Directly inhibiting caspase-1 activity can also block IL-1β/IL-18 maturation and pyroptosis. Rosmarinic Acid (RA) is a natural water-soluble phenolic acid compound. Studies found that RA alleviates cisplatin-induced acute kidney injury (CP-AKI) in mice, protects renal tubules, and improves renal function by blocking caspase-1 activation and inhibiting the NLRP3 inflammasome and upstream HMGB1-TLR4/MyD88 and NF-κB pathways (130). VX-740 and VX-765 are two types of peptidomimetic prodrugs that inhibit caspase-1. In models of rheumatoid arthritis and skin inflammation, VX-765 has been shown to alleviate disease severity and diminish the expression of inflammatory mediator (131). Nevertheless, the application of these two drugs in kidney disease, particularly AKI, has not yet been reported in clinical trials.

AC-YVAD-CMK is an irreversible caspase-1 inhibitor. Studies have confirmed that AC-YVAD-CMK can significantly downregulate caspase-1 activation and expression, inhibit GSDMD-mediated pyroptosis, and exert significant renoprotective effects in the S-AKI model of rats (132). Baicalin, extracted from the dried root of Scutellaria baicalensis and is the most abundant and representative flavonoid active monomer in Scutellaria. Research showed that baicalin alleviates iohexol-induced injury in HK-2 cells by inhibiting the ROS/NLRP3/caspase-1/GSDMD pathway-mediated pyroptosis by, which offers an experimental foundation for natural target drug candidates for CI-AKI (115). Cordycepin, the core nucleoside active monomer in Cordyceps sinensis, is primarily sourced from the fruitbody and mycelium of Cordyceps sinensis and Cordyceps militaris. Liu Z et al. demonstrated that cordycepin could inhibit the ROS/XO pathway and directly bind to and block the NLRP3/Caspase-1/GSDMD pathway, significantly reducing LPS-induced macrophage pyroptosis and the release of inflammatory factor, providing experimental basis for the targeted therapy of AKI (133).

### 5.5 Drugs targeting NLRP3 indirectly

In addition to specific inhibitors that directly block NLRP3 inflammasome assembly and activation, a variety of clinically available drugs have been shown to confer renoprotective effects inAKI through indirect modulation of NLRP3 signaling pathways. Representative agents include Roxadustat, Remdesivir, and Dexmedetomidine.

Roxadustat, as a hypoxia-inducible factor prolyl hydroxylase inhibitor, stabilizes HIF1A (hypoxia-inducible factor 1α) to improve the renal hypoxic microenvironment. It upregulates autophagy-related proteins including BECN1, BNIP3, and LC3B-II, promotes mitophagy, reduces ROS release, and blocks the ROS-TXNIP pathway, thereby decreasing NLRP3 assembly and activation and indirectly inhibiting NLRP3 inflammasome-mediated cell apoptosis (51). This mechanism has been particularly well established in contrast-induced AKI (CI-AKI) models and holds promise for extension to other AKI subtypes.

Remdesivir is a broad-spectrum antiviral nucleotide prodrug that does not directly bind to or inhibit the NLRP3 protein. Instead, it exerts renoprotective effects in acute kidney injury (AKI) by indirectly modulating upstream signaling pathways to downregulate NLRP3 activation. Remdesivir effectively suppresses the activity of nuclear factor-κB (NF-κB) and mitogen-activated protein kinases (MAPK), thereby reducing the transcription of NLRP3 inflammasome-related genes. Both *in vivo* and *in vitro* experiments have demonstrated that Remdesivir limits NLRP3 inflammasome activation (134), suggesting its potential therapeutic value for AKI patients in clinical settings.

Dexmedetomidine (DEX), a highly selective α2-adrenergic receptor (α2-AR) agonist, exerts renoprotective effects in septic kidney injury models by indirectly inhibiting NLRP3 inflammasome activation. Mechanistically, DEX activates α2-AR, promotes AMP-activated protein kinase (AMPK) phosphorylation, and suppresses mammalian target of rapamycin (mTOR) activity, thereby significantly enhancing autophagy in renal cells. The augmented autophagy facilitates the clearance of damaged intracellular organelles, which in turn suppresses NLRP3 inflammasome assembly and activation. Notably, pharmacological intervention with the autophagy inhibitor 3-MA or the α2-AR antagonist atipamezole significantly reversed the inhibitory effects of DEX on the NLRP3 pathway and its renoprotective effects, further confirming that α2-AR/AMPK/mTOR-mediated autophagy activation constitutes the core pathway through which DEX indirectly regulates the NLRP3 inflammasome (135).

In summary, a variety of drugs targeting the NLRP3 inflammasome or its upstream/downstream molecules have demonstrated protective effects in AKI animal models. These include direct inhibitors (e.g., MCC950, OLT1177, and natural products), mitophagy inducers (e.g., MCL and berberine), K^+^ efflux inhibitors (e.g., BHB and glyburide), caspase-1 inhibitors (e.g., rosmarinic acid, AC-YVAD-CMK, baicalin, and cordycepin), and indirect targeting agents (e.g., Roxadustat, Remdesivir, and Dexmedetomidine). However, the clinical translation of these agents remains confronted with numerous challenges, including issues related to specificity, safety, pharmacokinetic properties, and differential efficacy across AKI of various etiologies. Future research should focus on optimizing drug design and conducting more extensive large-animal studies and clinical trials, with the goal of providing safe and effective targeted therapeutic strategies for AKI patients.

## 6 Conclusions and future perspectives

AKI is a critical clinical condition driven by various etiologies such as ischemia, sepsis, drugs, or contrast agents. It is characterized by high incidence rate, a tendency to progress into CKD and ESRD, and the limitation of existing treatment approaches. In recent years, as research on the inflammatory mechanism of innate immunity has deepened, the NLRP3 inflammasome has gradually emerged as a key regulatory molecule in the field of kidney injury. This review provides a comprehensive account of the molecular assembly and the canonical and non-canonical activation cascades of the NLRP3 inflammasome. Notably, it presents, for the first time, a systematic synthesis of the PTMs regulatory networks—encompassing ubiquitination, phosphorylation, SUMOylation, and acetylation—and their contextual linkage to etiology-specific regulatory mechanisms in AKI. In addition, the review places particular emphasis on dissecting the divergent pathophysiological roles of the NLRP3 inflammasome across three major AKI subtypes: IR-AKI, S-AKI, and CI-AKI. The abnormal activation of the NLRP3 inflammasome in renal tubular epithelial cells and renal interstitial macrophages induces Gasdermin D-mediated pyroptosis through the release of IL-1β/IL-18, which amplifies the local inflammatory responses, promotes tubular necrosis and interstitial fibrosis, and ultimately accelerates the progression from AKI to CKD. Based on this, we reviewed the protective effects of strategies such as direct targeting NLRP3, regulating of mitophagy, intervention in with K^+^ efflux, and inhibition of caspase-1 in AKI animal models, highlighting the potential of the NLRP3 inflammasome as a therapeutic target for AKI. Meanwhile, it also reveals obvious limitations in current research regarding clinical translation, cell-type specificity, and long-term safety and efficacy validation.

Although a substantial number of breakthroughs have been achieved, numerous unsolved issues still persist in the research on the NLRP3 inflammasome in AKI. It is believed that future research should concentrate on the following aspects: 1. Elucidating the cell type-specific role of the NLRP3 inflammasome in AKI. Current evidence suggests significant differences in activation thresholds, downstream effects, and regulatory mechanisms of NLRP3 among different cell types such as renal tubular epithelial cells, macrophages, and mesangial cells. Future studies should employ single-cell sequencing, spatial transcriptomics, and conditional gene knockout techniques to precisely map the NLRP3 activation landscape in various renal resident and immune cells across different etiologies of AKI, thereby laying the foundation for developing cell-targeted intervention strategies. 2. Exploring the dynamic regulatory networks of NLRP3 PTMs. Various PTMs, including ubiquitination, phosphorylation, acetylation, and palmitoylation, play a precise regulatory role in NLRP3 activation. However, current research on the dynamic changes of NLRP3 PTMs under AKI pathological conditions remains limited. Future research should combine proteomics techniques to systematically identify the key PTM sites and regulatory enzymes of NLRP3 in AKI, reveal their spatiotemporal evolution, and propose novel intervention strategies targeting PTMs. 3. It is necessary to pay attention to the long-term effects of NLRP3 in the AKI-to-CKD transition. Currently, the majority of studies concentrate on NLRP3 activation during the acute phase of AKI, with inadequate attention to sustained low-level activation and fibrosis - driving role of NLRP3 in the chronic repair phase. Future research should establish continuous AKI-CKD models, dynamically monitor the relationship among NLRP3 inflammasome activity, renal tubular epithelial cell transdifferentiation, and fibroblast activation, and develop dual-targeting strategies that simultaneously block acute inflammation and chronic fibrosis.

In conclusion, the NLRP3 inflammasome represents a potential target for AKI intervention, however, it remains a considerable distance from clinical application. In the future, through more in – depth mechanistic analysis, more efficient drug design, and more rigorous clinical validation, the basic research findings should be genuinely translated into novel diagnostic and therapeutic strategies that reduce mortality and chronicization rates in AKI patients. We anticipate that drugs precisely targeting the NLRP3 inflammasome will enter the clinical practice of nephrology as soon as possible, thereby bringing substantial benefits to the vast majority of patients with acute kidney injury.
